# Decoding Allosteric Inhibition in MALT1: The Hidden Role of Conformational Plasticity in Metastable States via Biased MD and Deep Learning

**DOI:** 10.1021/acs.jpcb.5c07665

**Published:** 2026-01-20

**Authors:** Rodrigo M. Santos, Taináh M. R. Santos, Teodorico C. Ramalho

**Affiliations:** † Laboratory of Molecular Modelling, Department of Chemistry, Federal University of Lavras, Lavras 37200-000, Minas Gerais, Brazil; ‡ Centre for Basic and Applied Research, Faculty of Informatics and Management, University of Hradec Králové, Hradec Králové 500 03, Czech Republic

## Abstract

The mucosa-associated lymphoid tissue lymphoma-translocation protein 1 (MALT1) is a key protein in the adaptive immune response system in humans. This protein is widely expressed in the human body and is related to nuclear factor-κB (NF-κB) signaling activation in response to T-cell receptors. Due to this, MALT1 is key in the regulation of inflammatory events in a variety of tissues, where its dysregulation is associated with several types of cancer, especially hematological cancers. In this sense, its relevance makes MALT1 a valuable target to treat many diseases, drawing the attention of many researchers with the aim of proposing new MALT1 inhibitors. However, there is a lack of literature describing its complex dynamical behavior and allosteric inhibition, which considerably hampers the computational design of new MALT1 allosteric inhibitors. In that regard, the present work investigated the complex conformational behavior of MALT1 protein during its allosteric inhibition. For this, biased molecular dynamics simulations, sophisticated machine learning techniques such as neural networks, and docking calculations were used. From the performed investigation, it was observed that through allosteric inhibition, Loop 1 and 3 movements were crucial to reduce the catalytic site cavity volume, keeping cysteine unavailable for substrate mimic binding. In addition, statistical information over the explored ensemble showed that the great majority of the inhibited conformations presented an unavailable catalytic cysteine for substrate binding. Hence, the presented results can be used as an objective criterion for the computational proposal of new MALT1 allosteric inhibitors. However, despite mouse MALT1 and human MALT1 presenting 93% homology, the generalization of the findings to human MALT1 protein should be taken with care, and the obtained results apply specifically to the mouse MALT1 construct.

## Graphical abstract

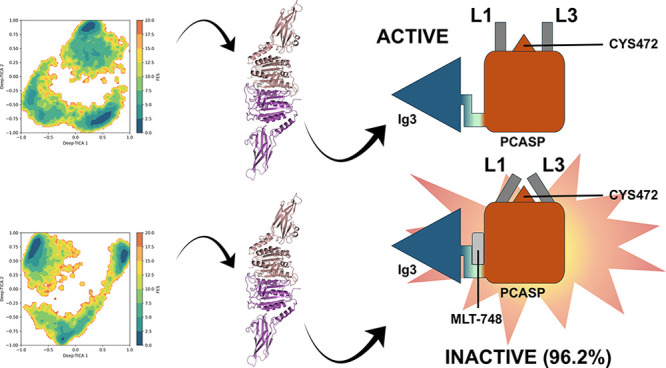

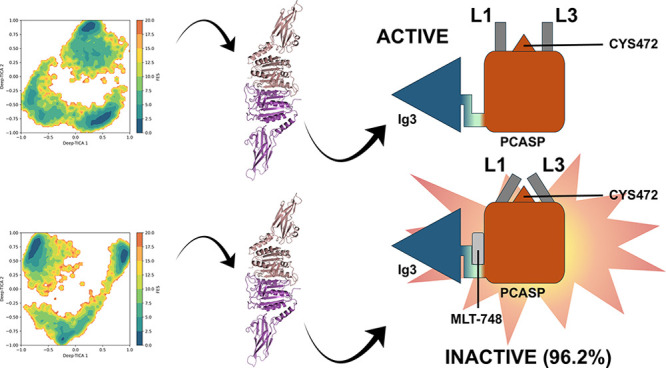

## Introduction

One protein that has drawn much attention from researchers in human adaptive immune responses is the mucosa-associated lymphoid tissue lymphoma-translocation protein 1 (MALT1), a unique cysteine caspase-like protein that is widely expressed in the human body.^1–4^ MALT1 is a key protein in the nuclear factor-κB (NF-κB) signaling activation, required to regulate lymphocyte proliferation and activation, immune responses, and regulating inflammatory events in many tissues.^1,5–8^

Due to its key role in the adaptive immune response, MALT1 dysregulation is associated with a variety of types of cancer, such as hematological cancers, breast cancer, and melanoma.^6,9,10^ In fact, chronic MALT1 activation has also been related to the survival of solid tumors, where previous studies showed its critical function in Treg cells in order to maintain an immunosuppressive tumor microenvironment in these solid tumors.^9,11,12^ In addition, there are studies showing the importance of MALT1 in neurological functions, where neuroinflammation, a condition present in a series of neurodegenerative diseases like Alzheimer’s and Parkinson’s disease, can be reduced through MALT1 inhibition.^13,14^ Hence, with such a critical role in the human immune system and its relationship to a variety of anomalies, MALT1 shows itself to be a relevant target to potentially treat many diseases.

Despite its expressive relevance in many diseases, MALT1 protein is not an easy target for drug proposal, since its complex structure and conformational behavior substantially hamper such a goal. MALT1 is composed of several domains, including three immunoglobulin (Ig) domains, one N-terminal death domain (DD), and a paracaspase domain (PCASP), containing the cysteine catalytic site.^5,15^ With such a complex structure, MALT1 presents intrinsically disordered regions, where activation relies on the stabilization of disordered loops at the substrate-binding pocket, usually upon ligand binding.^16^

In this scenario, investigating proteins with intrinsically disordered regions to understand the inhibition mechanism as well as propose new inhibitors is of great complexity, since this regions does not follow the rule of a unique three-dimensional structure per amino acid sequence.^17,18^ Therefore, due to the fact that not only MALT1 presents disordered regions, but also a series of biochemical processes that involves MALT1 occurs between intrinsically disordered structures, it makes a challenge to investigate its inhibition mechanisms and related protein movements.^16,19,20^ Hence, the dynamical study of disordered proteins such as MALT1 is of utmost value, where fancy techniques such as molecular dynamics and machine learning can be up to this challenge, helping to unravel the complex conformational pattern of this regions during MALT1 inhibition.^21–23^

Although the proposal of MALT1 inhibitors is of great complexity due to the disordered nature of some loop regions, previous works have dedicated efforts in this direction. With the aim of developing an effective MALT1 inhibitor, Rebeaud et al. developed the first irreversible peptide-based inhibitor by mimicking the substrate that binds to the cysteine catalytic site, which is the Z-VRPR-fmk inhibitor.^15,24^ However, one interesting fact about Z-VRPR-fmk inhibition is that it occurs solely in a competitive way, not preventing the PCASP domain of MALT1 from being in an active conformation, where Z-VRPR-fmk binding also induces dimerization, an important step in MALT1 activation.^5,15,25^

In that regard, another interesting strategy for MALT1 inhibition relies on the development of allosteric inhibitors, comprising an allosteric pocket localized between the PCASP-Ig3 domains, capable of inhibiting the protein in a noncompetitive way.^26–28^ From this strategy, the aim is to stabilize an inactive PCASP domain by inducing allosteric conformational changes in MALT1, where previous studies have shown a promising inhibition potency for some allosteric inhibitors, like MLT-748.^27–29^ Despite important experimental results regarding the potency of proposed allosteric inhibitors being obtained, to the best of our knowledge, there is a lack of literature describing the dynamical behavior of such a PCASP inactivation, a problem that was also pointed out in the recent study developed by Wallerstein et al.^30^ With this absence of knowledge, the proposal of new allosteric inhibitors has turned out to be a challenge in computational drug design.

Moreover, MALT1 protein activation and inactivation comprise a series of conformational shifts,^15,31^ also hampering the design of new allosteric inhibitors for MALT1. Therefore, due to MALT1’s conformational complexity, the comprehension of its metastable states ensemble can be of great value for understanding not only its dynamical behavior but also the statistical distribution of active and inactive conformational populations. In fact, the comprehension of metastable states and intermediate states can be an insightful strategy for the poorly understood mechanisms of proteins containing intrinsically disordered regions, such as the MALT1 protein.^32–34^

It is known that computational frameworks can be very insightful for investigating biological systems.^35–39^ In this sense, for such a computational investigation of metastable states, many studies have dedicated effort to developing enhanced sampling techniques to access these ensembles and analyze them, where the usage of sophisticated techniques, such as neural networks, is of great value in these biological investigations.^40–44^ Their usage ranges from understanding the role of water in complex systems involving protein inhibition, protein conformational behavior, and proposal of a new biological probe targeting a cancer-relevant protein.^45–47^ Therefore, their usage to unravel the conformational behavior in activation and inactivation of MALT1 can be of great value, and from our research, this is shown to be a novelty in the MALT1 literature.

In this scenario, the main goal of the present work is to investigate the conformational behavior in MALT1 inactivation through allosteric inhibition using the MLT-748 inhibitor, enhanced sampling techniques (biased molecular dynamics), and neural networks.

## Materials and Methods

### Protein and Ligand Structure Preparation

The initial structure of MALT1 used for theoretical calculations was the *Mus musculus* active MALT1 (PDB: 3V4L),^25^ as it presents no missing residues in its structure and almost fully crystallized disordered regions. This is of great importance, since completing residues can drive the structure to nonphysical conformations, and to the best of our knowledge, there is a lack of inactive MALT1 crystals with no missing residues, which prevents their usage as an initial structure. Therefore, with the collected crystal, the irreversible covalent inhibitor Z-VRPR-fmk was removed in order to obtain a clean monomer of MALT1.

The *Mus musculus* MALT1 presents an identity of 93% when compared to human MALT1 (PDB: 3V55),^25^ as can be seen in Figure 1a, and the same key residues in the catalytic site, as can be seen in Figure 1b. Hence, the high similarity between the sequences allows an appropriate comprehension of the dynamic behavior of human MALT1 during allosteric inhibition using the *Mus musculus* MALT1 for theoretical findings. In this scenario, to improve clarity and comparability between the MALT1 species, Figure 1b shows the key catalytic site residues for both, being Cys464–Arg465–Lys466–Arg467 in human MALT1, and Cys472–Arg473–Lys474–Arg475 as their equivalents in *Mus musculus* MALT1.

**Figure 1:**
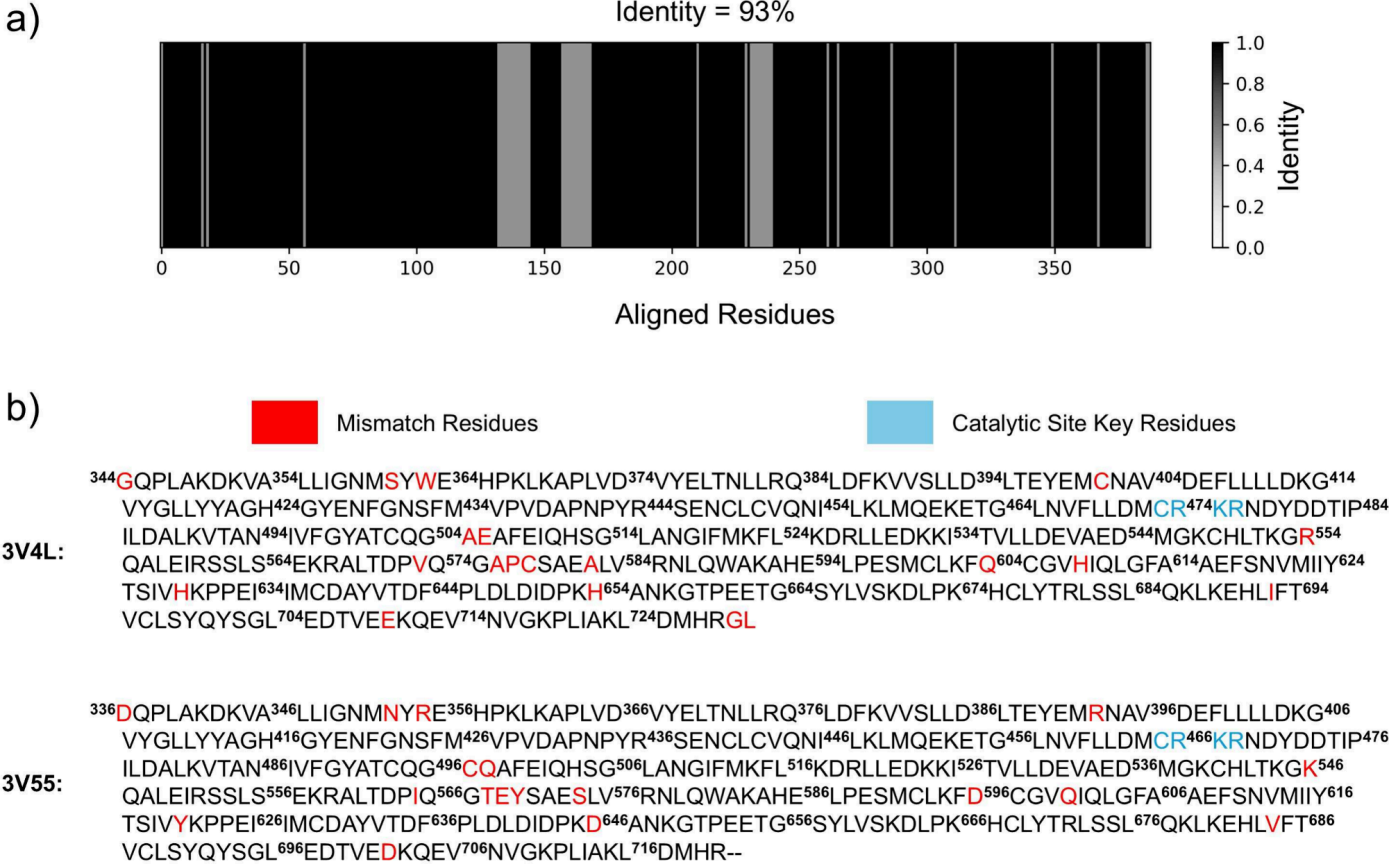
Sequence alignment between *Mus musculus* MALT1 (PDB: 3V4L)^25^ and human MALT1 (PDB: 3V55).^25^ (a) Shows the high similarity of 93% between the sequences and (b) shows the sequences of both species with their respective numbering, highlighting the mismatched residues and the catalytic site key residues.

Now, for allosteric inhibition investigation, the ligand used was MLT-748, an allosteric inhibitor whose structure was collected from PDB: 6H4A.^29^ The MLT-748 allosteric inhibitor showed great inhibitory activity, being appropriate for the scope of investigation in the present work.^29^ To complete the hydrogens of the collected structure, Avogadro 1.2.0 ^48^ was used, and to generate the topology, the CGenFF web server^49^ was used. In order to correctly position MLT-748 in the clean MALT1 monomer, the processed 3 V4L structure was aligned with the 6H4A structure (containing MLT-748 at the allosteric pocket), and then, the ligand was positioned in the clean 3V4L monomer.

### Unbiased Molecular Dynamics (MD) Simulations Setup

Six unbiased MD simulations of 200 ns each were performed, being three replicas of the clean MALT1 monomer in water (system 1, noninhibited system), and three replicas of the MALT1 monomer with MLT-748 positioned in its allosteric binding site, also in water (system 2, inhibited system). For the simulations, GROMACS 2023.3 ^50^ was used, following the steps of energy minimization, NVT equilibration, NPT equilibration, and the unbiased production. For describing the system’s potential, the CHARMM36 force field^51^ was used, and for the water model, SPC was used.

Before proceeding with the minimization step, the built system’s charge was neutralized once it presented a nonzero total charge of −13, in which 13 sodium ions were added to the system. Now for energy minimization, in this step, the steepest descent integrator was used with a time step of 5 fs, where minimization followed until maximum force converged to values lower than 1000 kj/mol. Further, the minimized system was submitted to NVT equilibration during 100 ps, where leapfrog integrator was used with a time step of 2 fs, and this equilibration step was performed at 300 K. In the NVT equilibration step, the replicas were generated for each system by changing the velocities generation seeds, using as seeds −1, −2, and −3 to differentiate starting conditions of the three replicas, where the mentioned seeds were used for both system 1 and system 2 replicas. With the NVT equilibrated system, NPT equilibration was performed also during 100 ps, using the same integrator and time step. In addition, in this step, Berendsen was used for pressure coupling and a velocity rescaling thermostat for temperature coupling.

With the minimized and equilibrated systems, it was possible to produce 200 ns of unbiased MD simulations. In the configuration of the unbiased production step, a time step of 2 fs was used with the leapfrog integrator. In addition, Parrinello–Rahman with isotropic pressure coupling was used with a compressibility factor of 4.5 × 10^–5^, and for temperature coupling, velocity rescaling thermostat was used. Now, for neighbor searching and van der Waals interactions, the Verlet Cutoff scheme was used with a cutoff of 12 Å, and for long electrostatic interactions, Particle Mesh Edwald (PME) was used with the same cutoff.

### Biased Molecular Dynamics (MD) Simulations Setup

Now, from biased MD simulations, it was possible to access the metastable states of MALT1 of both systems described in the unbiased procedure. In this sense, for the biased simulations, GROMACS 2023.3 ^50^ patched with PLUMED 2.9.0 ^52^ was used. Therefore, two biased MD simulations of 200 ns were performed, one for the noninhibited system (system 1) and one for the inhibited system (system 2). The biased MD simulations also followed the steps of energy minimization, NVT equilibration, NPT equilibration, and biased production. For describing the system’s potential, the CHARMM36 force field^51^ was also used with the SPC water model.

The same setup used in the unbiased MD simulations was used in the biased MD simulations for all of the performed steps, with an additional step of biasing in the biased production. For the production, the used biasing technique was the on-the-fly probability enhanced sampling (OPES) expanded.^40^ In this sense, a multithermal expansion collective variable was used, where the potential energy *U* was biased in order to target the multicanonical ensemble over a temperature range of 300–400 K. In a scenario where the metastable states of the investigated systems are unknown, biasing the potential energy *U* can be a good strategy, avoiding the choice of poor collective variables.^40^ The pace of the bias potential update was set to 500 simulation steps (1 ps).

### DeepTICA Analysis for Conformational Landscape Obtention

In order to identify and characterize the metastable states of the investigated MALT1 systems, hydrogen bond contact distances were calculated by using the Python library md-stateinterpreter^41^ and used as the set of physical descriptors for further analysis. In addition, since MALT1 is a large protein, it was needed to filter the obtained descriptors in order to reduce the data set dimensions and create a more computationally feasible data set. Hence, it was maintained in the data set only the hydrogen bond contact distances that formed an hbond in at least 10% of the frames, considering that the hbond is formed if the distances are lower than or equal to 3.5 Å. Moreover, since the present work’s main goal is to achieve a comparison between the simulated systems, it was necessary to ensure that both data sets of system 1 and system 2 contain distance information on the same hydrogen bond contacts. In this sense, the hydrogen bond contacts that were not present in both system 1 and 2 data sets were discarded. From this procedure, a data set of 602 hydrogen bond contact distances was obtained for both systems.

Once the appropriate set of physical descriptors is obtained, it is possible to build a conformational landscape of the metastable states accessed from biased MD simulations. For that, the DeepTICA method^53^ can be used once it is capable of differentiating the metastable states in a clustering procedure that can be observed in only two dimensions, which facilitates visual identification and interpretation, also allowing a population analysis of these metastable states.

This is done by using the Variational Approach to Conformational Dynamics (VAC),^54,55^ which focuses on the transfer operator $T_{\tau }$ evolving the probability density *u*_*t*_ by a time τ toward the equilibrium Boltzmann distribution, following the equation below:^41,53^

$$u_{t+\tau }\left(R\right)=T_{\tau }◦u_{t}\left(R\right)$$

with **R** being a provided set of physical descriptors as a function of the atomic coordinates. This equation is of singular interest once the first eigenfunctions identify modes that relax more slowly toward the equilibrium, which enables metastable states identification.^41^ Despite that, solving this equation can be challenging; however, since the eigenfunctions are maximally autocorrelated, its eigenfunctions can be obtained with Time-lagged Independent Component Analysis (TICA),^54,56^ a statistical method that searches for the linear projection of a set of descriptors with maximum autocorrelation.^41^ In this scenario, with the help of artificial neural networks (ANN), the eigenfunctions can be obtained by training the ANN to maximize the TICA eigenvalues,^41^ this procedure being called DeepTICA.

In this sense, a DeepTICA analysis was carried out for the noninhibited and inhibited MALT1 systems. For that, the Python libraries mlcolvar 1.1.1,^57^ pytorch 2.2.2,^58^ and lightning 2.4.0 ^59^ were used. For the DeepTICA analysis, [602,30,30,2] was used as the neural network architecture, with the first 602 nodes corresponding to the 602 hydrogen bond contact distances used as physical descriptors, 30 nodes for each of the two hidden layers, and 2 nodes for the output layer, which gives the two DeepTICA collective variables (CVs). From these two DeepTICA CVs, it is possible to obtain a two-dimensional conformational landscape that facilitates interpretation and labeling. In addition, the first 50 ns of the simulations were discarded in order to ensure that only the bias-converged part of the simulations was used for the analysis (see Figure S1 for bias profile). Furthermore, 20% of the data set was separated for validation during training, and a lag time of 1.0 was used.

### Modeling the MALT1 Dimer Interface: Insights from Protein–Protein Docking

Regarding the performed DeepTICA analysis, it was possible to select representative conformations of the accessed metastable state of each investigated system for further structural analysis. Therefore, with the selected conformations, a protein–protein docking was performed in order to build an MALT1 dimer, for which the ClusPro web server^60,61^ was used. In order to correctly create the dimer, attractions were set between the amino acid residues that are present in the dimer interface, being the residues 428–429 (corresponding to the β_3*A*/*B*_ portion), residues 473–493 (corresponding to the Loop 2 portion), and residues 529–567 (corresponding to the β_6_ portion). The dimer with the lowest energy score was selected as the correctly built dimer.

### Covalent Docking of a Substrate Mimic to the MALT1 Dimer

With the collected representative conformations, a covalent docking between the constructed MALT1 dimers and a substrate mimic was performed in order to evaluate the availability of CYS472 as an important catalytic site residue. In this sense, the substrate mimic Z-VRPR-fmk peptide covalent inhibitor was chosen, as its action relies on its competition with the substrate for the catalytic site of MALT1, covalently binding to the sulfhydryl group of the CYS472 amino acid residue. For the covalent docking, AutoDockFR (ADFR)^62^ was used.

For the covalent docking setup, the Z-VRPR-fmk structure was obtained from PDB: 3V4L^25^ and positioned in the constructed dimers by aligning the dimer structures with the crystal structure. After positioning the structure, the covalent bond was built and described for the target file creation in ADFR. The covalent atoms involved in the covalent bond are carbon C1 (from Z-VRPR-fmk) and sulfur SG (from MALT1 CYS472), where carbon CB of CYS472 was used as an anchor. Now the constructed box used the sulfur SG atomic coordinates as the center with grid dimensions of 35 Å in all directions. The lowest energy pose of the docking calculation was selected, being the most stable.

## Results and Discussion

### Unbiased MD Analysis of Monomeric MALT1

As an initial comprehension of the investigated systems, unbiased MD simulation trajectories were analyzed in terms of their number of hbonds performed by the MALT1 protein in both noninhibited (system 1) and inhibited (system 2) systems. The main objective of this analysis is to obtain relevant information about the difference in conformational plasticity regarding allosteric inhibition. Hence, Figure 2 shows the number of hbonds performed by MALT1 with itself and with the water present in the simulated chemical environment for all replicas of both systems.

**Figure 2:**
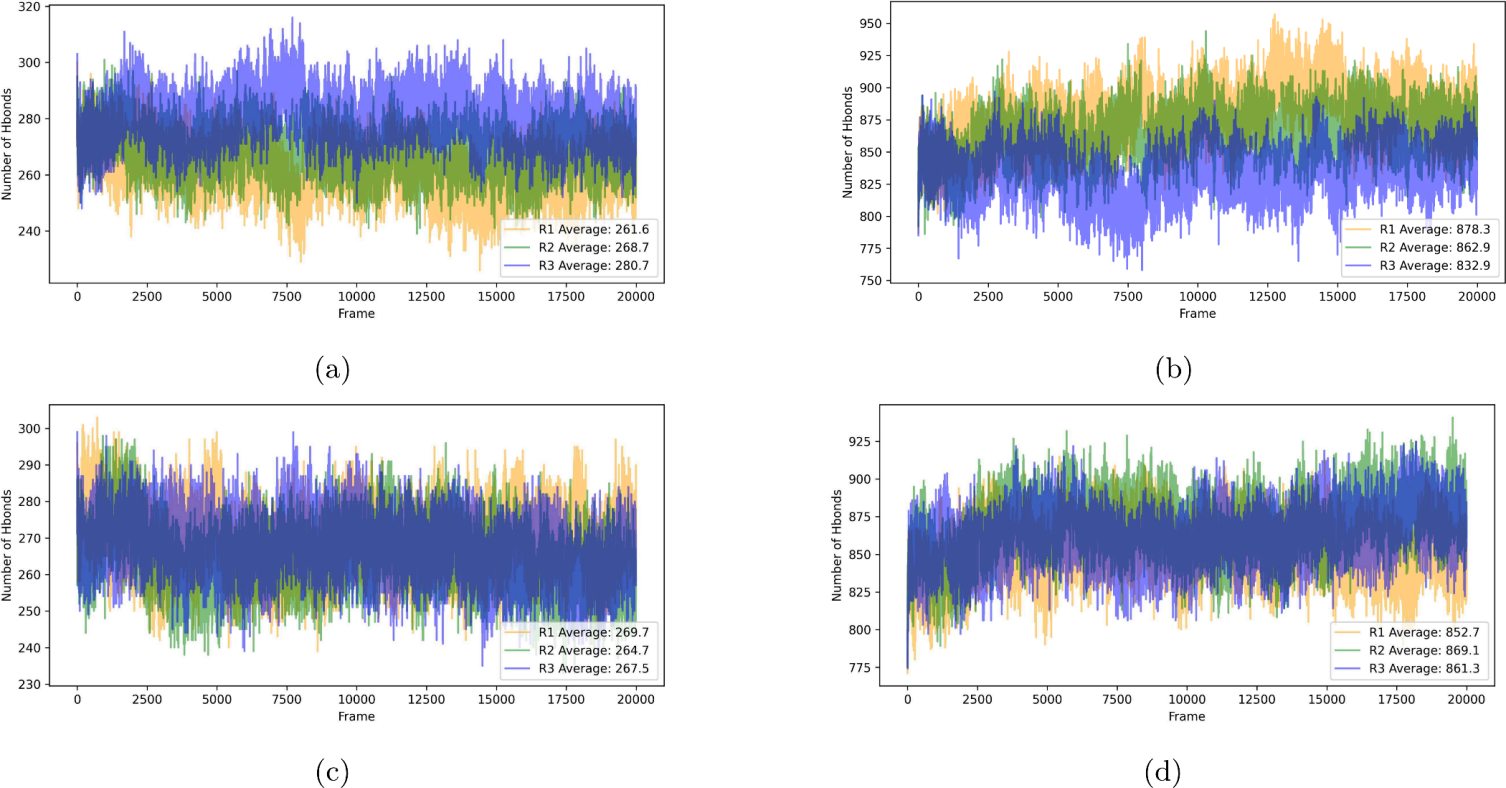
Total number of hbonds performed by MALT1 with itself in (a) system 1 and (c) system 2. In addition, the total number of hbonds performed by MALT1 with the water present in the chemical environment of (b) system 1 and (d) system 2.

For system 1, Figure 2a shows the total number of hbonds performed by MALT1 with itself, in which it obtained an average of 261.6 for replica 1, 268.7 for replica 2, and 280.7 for replica 3, with standard deviations of 8.7, 7.9, and 8.4, respectively. Therefore, for system 1, an overall average of 270.3 was obtained with an overall standard deviation of 7.9. Now considering system 2, Figure 2c shows the total number of hbonds performed by MALT1 with itself, obtaining an average of 269.7 for replica 1, 264.7 for replica 2, and 267.5 for replica 3, with standard deviations of 8.5, 8.4, and 8.1, respectively. For system 2, the overall average was 267.3, with an overall standard deviation of 2.0.

Now with respect to the hbonds performed between MALT1 and the water present in the chemical environment, Figure 2b shows the total number of hbonds for system 1. In this scenario, it was obtained an average of 878.3 for replica 1, 862.9 for replica 2, and 832.9 for replica 3, with standard deviations of 21.4, 18.4, and 18.2, respectively. The overall average for this system was 858.1, with an overall standard deviation of 18.8. Regarding system 2, Figure 2d shows that it obtained an average of 852.7 for replica 1, 869.1 for replica 2, and 861.3 for replica 3, with standard deviations of 17.6, 19.4, and 17.7, respectively. For system 2, the overall average was 861.0, with an overall standard deviation of 6.7.

Therefore, the obtained results show a considerable decrease in the overall standard deviation for system 2 when compared with system 1 in both hbond analyses. This is the result of a reduction in MALT1 degrees of freedom after positioning the MLT-748 allosteric inhibitor, showing that the allosteric inhibitor generates a more stable evolution of MALT1. In addition, RMSD analysis of the unbiased MD replicas (see Section SI.2 for details) also supports this conclusion, with a decreased overall standard deviation for system 2.

In this scenario, the impact of allosteric inhibition in MALT1 evolution observed through unbiased MD simulations from this, it is not possible to observe conformational plasticity patterns of MALT1 inhibition. This occurs once unbiased MD simulations perform a limited exploration of the conformational landscape of the investigated system. Hence, for an appropriate exploration, the usage of biased MD simulations was needed, with this technique being capable of enhancing MALT1 fluctuations and accessing the stabilized metastable states. Therefore, with the accessed metastable states, sophisticated machine learning techniques can be used to analyze the hidden patterns of MALT1 disordered loops and obtain crucial insights into allosteric inhibition mechanism insights.

### Monomer Metastable States Identification and Clustering Behavior

The intrinsically disordered regions of MALT1 play a critical role in its allosteric inhibition mechanism, making the system particularly challenging to investigate both experimentally and computationally. In this context, the use of biased molecular dynamics in combination with machine learning techniques provides a powerful framework for probing the conformational plasticity of these disordered regions during the inhibition process. That said, the metastable states of MALT1 in water (system 1) and inhibited MALT1 in water (system 2) were first observed and identified from the construction of the DeepTICA conformational landscape by using biased MD trajectories. From such analysis, it was possible to observe how the metastable states of both systems were distributed and differentiated. In addition, after the metastable state identification, representative conformations of the observed metastable states could be collected for further structural analysis.

In that regard, it is possible to observe from Figure 3 that both systems presented a similar distribution of the accessed metastable states in which three different metastable states could be observed in each system, indicated by the three basins of higher point density. In order to observe the distribution with the associated computed FES and the obtained learning curves during training, see Figures S2 and S3. From this observation, the identified basins were labeled in both systems, and a Multilayer Perceptron (MLP) Classifier was used to validate the labeling and ensure that the observed metastable states could be differentiated from one another. The labeling validation step is of great importance, as it provides the information that basins contain structural information capable of differentiating them from other conformational metastable states.

**Figure 3:**
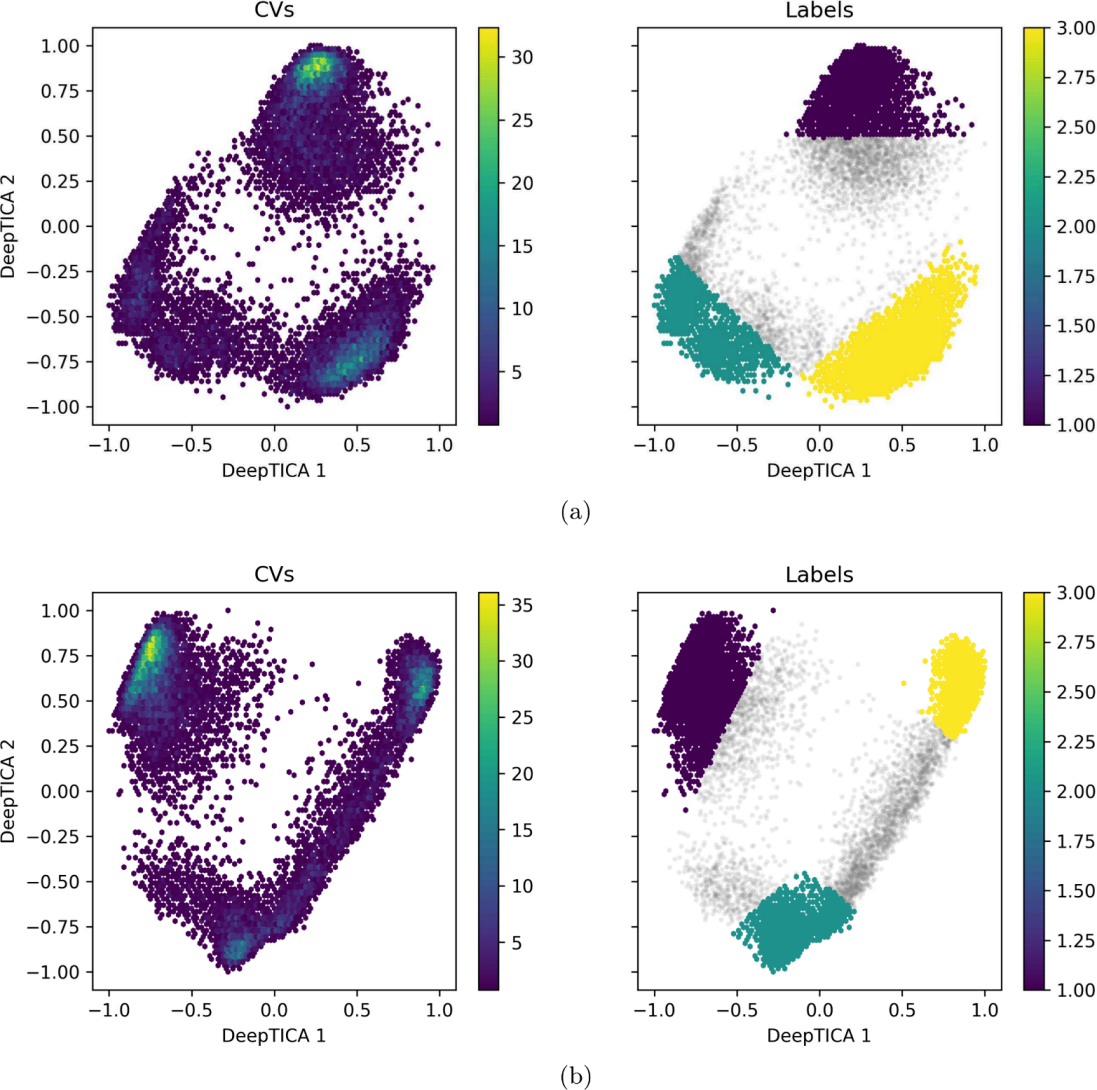
DeepTICA conformational landscape density plot with the performed labeling of (a) system 1 and (b) system 2. In the labels plot, the gray points indicate non-labeled data and the colored points indicate labeled data as numbers, according to the right positioned colorbar.

The MLP Classifier took as inputs the same hydrogen bond distances that were used to build the DeepTICA conformational landscape; therefore, the architecture of the classifier followed as [602,100,100,100,3] (Figure S6). From this architecture, 1000 samples were separated from the data set to be tested after training, and 20% of the trainable data set was separated for validation during training (for details about MLP setup and training, see Section SI.4). The MLP Classifier was built and trained using Tensorflow 2.17.0,^63^ and the same architecture and train-test split were used for both systems labeling validation.

For the 1000 samples separated for testing after training, with the correctly trained neural network, the 1000 samples prediction presented a 100% accuracy for both systems, showing great generalization and label prediction capability. Therefore, the presented results show that the labels were correctly assigned in both systems and that the metastable states contain relevant conformational structure information capable of differentiating one metastable state from another.

In this scenario, the trained MLP Classifiers of both systems were also used to predict the separated 1000 sample labels of the other system. In this sense, the trained MLP Classifier of system 1 was used to predict the labels of system 2 samples and the MLP Classifier of system 2 was used to predict the labels of system 1 samples. This strategy is of great value once it is capable of providing information about the correlation of metastable states between systems, showing if the observed metastable states of the systems are equivalent or are different. Hence, by using the MLP Classifier of system 1 to predict system 2 samples, an accuracy of 19.9% was achieved (see Figure S8), and by using the MLP Classifier of system 2 to predict system 1 samples, an accuracy of 25.9% was achieved (see Figure S9). The results show a poor accuracy for both predictions, indicating that the observed metastable states in the noninhibited system (system 1) are different from the ones observed in the inhibited system (system 2). Therefore, by positioning the allosteric inhibitor in MALT1, different metastable states are accessed and the observed conformational landscapes are not equivalent between systems.

Moving further, with validated labeling, it is possible to perform a population analysis in order to observe the distribution of the accessed conformations over the observed metastable states in each system. Therefore, for system 1, it was observed that 5313 conformations belong to metastate 1, 1776 conformations to metastate 2, and 4936 conformations to metastate 3. Thus, metastable states 1 and 3 are capable of stabilizing many more conformations than metastable state 2, inferring greater stability for those metastable states.

Regarding system 2, 6997 conformations were observed in metastate 1, 2406 conformations in metastate 2, and 2511 conformations in metastate 3. Hence, in system 2, metastable state 1 presented a much higher population when compared to metastable states 2 and 3, which implicates a higher stability for this metastable state.

In light of the clustering behavior analysis, it was shown that MALT1 stabilizes three different metastable states in both noninhibited and inhibited systems. However, in the presence of an allosteric inhibitor, different metastable states are stabilized. Therefore, with the macrostructure information on the metastable states, representative conformations of each metastate in each system can be collected in order to observe the conformational structural features of the metastable states. For simplicity, metastable states will now be named as Meta “System number”.”Metastate label” in order to make it clear for the reader to which metastable state of which system the text is referring to.

### Exploring the Conformational Landscape of Monomeric Metastable States

The comprehension of the structural patterns that stabilize the observed metastable states is of great value in order to obtain mechanistic insights into allosteric inhibition. However, such an evaluation is of great complexity and needs a careful analysis of the structures that populate the accessed metastable states. Therefore, initial RMSD analysis of the biased MD simulations showed that the accessed structures of system 2 deviate more from the crystal active structure (see Section SI.5), which is expected. However, the present work’s main goal is to deeply understand structural patterns related to allosteric inhibition, in which a closer look at the accessed structures is necessary.

In this sense, to understand structural features of the accessed metastable states, representative conformations were selected to make this inspection feasible and allow pattern recognition over the structures. To select representative structures, the conformation nearest the center of mass of each cluster identified in the DeepTICA conformational landscape was used. This procedure is similar to the one previously developed in the recent work published by Santos et al.,^47^ which is very useful to analyze conformational patterns from clustering landscapes. In total, six conformations were collected, one for each metastate of its respective system. In addition, to make a clear and relevant comparison, the inspection focused on the catalytic site of MALT1, a region located around CYS472 on the caspase domain of MALT1. Previous studies have shown that the availability of CYS472 is crucial for MALT1 activity once the substrate binds in this amino acid residue.^5^

Starting with the inspection of system 1 selected conformations, Figure 4 shows a closer view of the catalytic sites of the collected conformations, emphasizing Loop 1 (L1) and Loop 3 (L3) positions and also the CYS472 position, shown as sticks. It was possible to observe for meta 1.1 and 1.3 (Figure 4a and Figure 4e, respectively) an open L3 conformation in a position that does not cover CYS472 and an L1 bending toward the opposite direction of the catalytic site, also not hampering CYS472 availability. It is important to mention that L1 is more bent in meta 1.1 than in meta 1.3. Now, for meta 1.2 (Figure 4c), a more closed L3 conformation was observed, which makes its left side closer to L1, which is also bending toward the opposite direction of CYS472; however, it is not bending as much as observed for meta 1.1 and 1.3. In addition, despite meta 1.2 showing a more closed L3 conformation, it does not seem to make CYS472 more unavailable.

**Figure 4:**
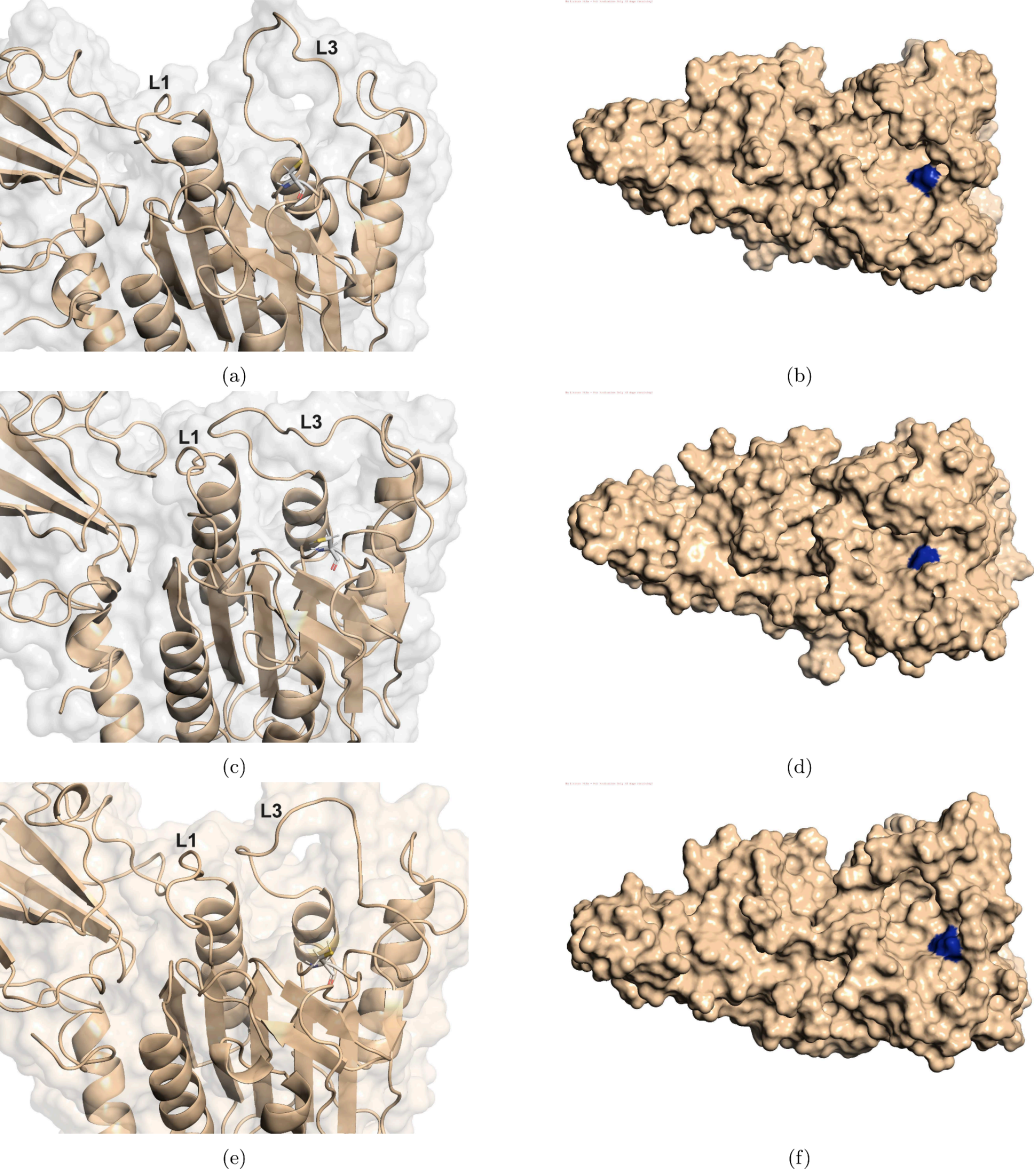
Shows the catalytic site of MALT1 protein of the collected structures from system 1, emphasizing Loop 1 and Loop 3 (annotated as L1 and L3, respectively), and cysteine (CYS472), shown in sticks representation. (a and b) Meta 1.1, (c and d) meta 1.2, and (e and f) meta 1.3 in cartoon and surface representation, side by side.

In order to better observe the size of the cavity in the catalytic site and to obtain important information about CYS472 availability, surface representation is of great value. From Figures 4b, 4d, and 4f, it was possible to observe that all accessed metastable states of system 1 presented an appropriate cavity size, where CYS472 (represented in dark blue) can be easily seen in the cavity, showing it to be available for binding to the substrate.

From the mentioned inspection, it seems that system 1 was capable of stabilizing only metastable states with an appropriate cavity size. The obtained results show that in the absence of an allosteric inhibitor, the caspase domain of MALT1 stabilizes conformations appropriate for substrate binding. In addition, meta 1.1 conformations, which represent the majority part of the labeled conformations, presented a larger cavity size, also supporting the CYS472 availability of the noninhibited MALT1 conformations. Therefore, from system 1 conformations, it was possible to observe the crucial roles of L1 and L3 in maintaining an appropriate catalytic site environment for CYS472 availability.

Moving further to system 2, the inhibited system, Figure 5 shows a closer view of the catalytic site of the collected structures, emphasizing the same loops (L1 and L3) and displaying CYS472 as sticks. It was observed for meta 2.1 and 2.3 (Figures 5a and 5e) a more closed L3, while a much more open L3 was observed for meta 2.2 (Figure 5c). In addition, it was observed that in meta 1 and 3, the L3 left portion is bent toward the catalytic site, and in meta 2.2, a retreat of this left portion of L3 was observed. This L3 behavior observation is of great importance, indicating that meta 2.1 and 2.3 present a more closed catalytic site than meta 2.2.

**Figure 5:**
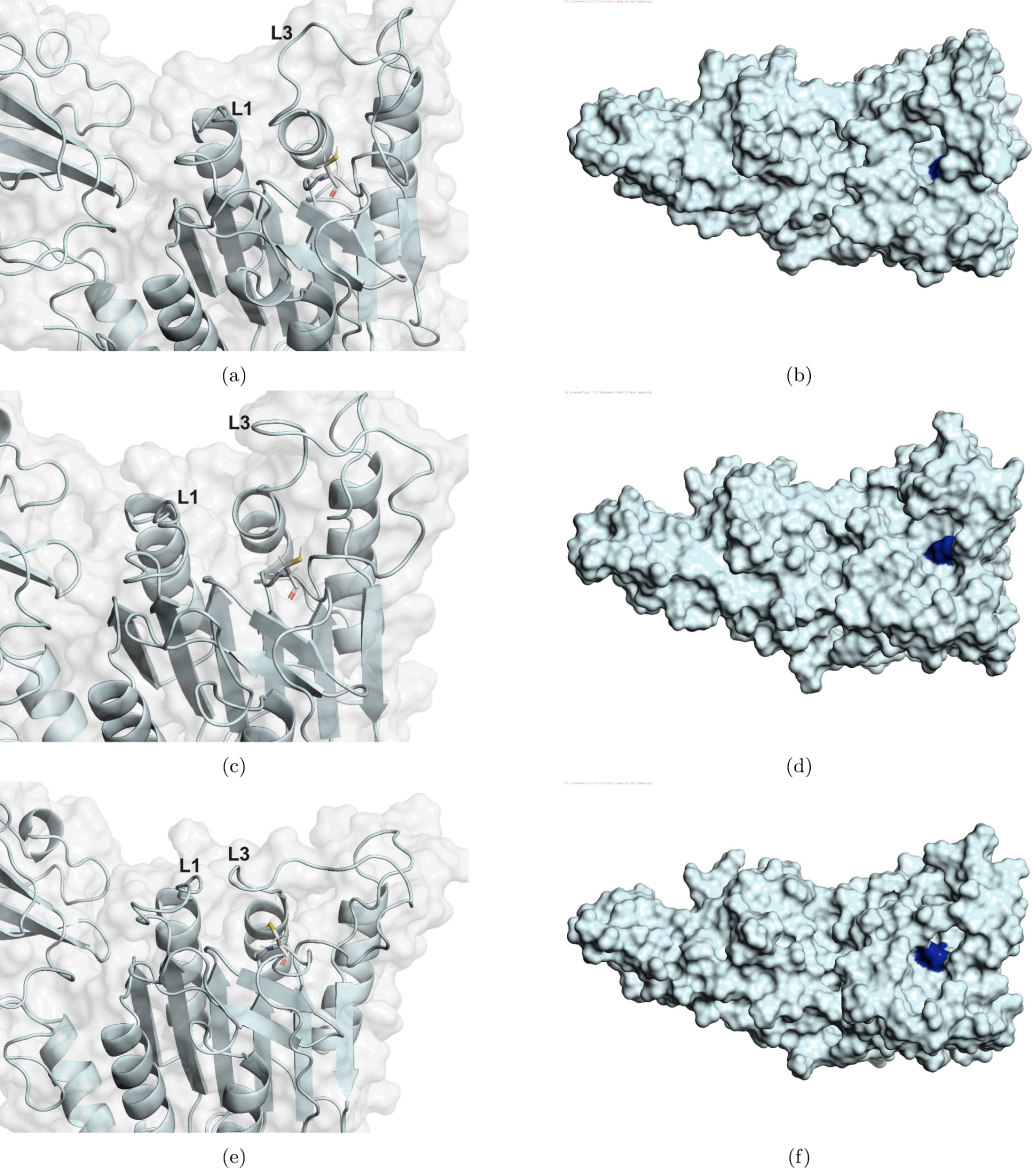
Shows the catalytic site of the MALT1 protein of the collected structures from system 2, emphasizing Loop 1 and Loop 3 (annotated as L1 and L3, respectively) and cysteine (CYS472), shown in sticks representation. (a and b) Meta 2.1, (c and d) meta 2.2, and (e and f) meta 2.3 in cartoon and surface representation, side by side.

Another important feature that was observed for the inhibited conformations is the L1 bending toward the catalytic site (the opposite movement that was observed for the noninhibited conformations of system 1). All inhibited conformations presented this movement, with it being more pronounced in meta 2.1 and 2.3 conformations (Figures 5a and 5e). In this sense, with the combined L1 and L3 movement toward the catalytic site of MALT1, a higher unavailability of CYS472 is expected. However, it is important to mention that, despite the described L1 movement also being present in the meta 2.2 (Figure 5c) conformation, the retreat in L3 movement and its chain opening are sufficient for not closing the catalytic site as observed for meta 2.1 and 2.3.

Now observing the surface representation of the inhibited conformations for a better comprehension of its catalytic site size, from Figures 5b and 5f it is possible to observe a much more closed catalytic site when compared to system 1 collected structures. The described L1 and L3 were capable of “hiding” CYS472 (represented in dark blue), making it more unavailable for substrate binding. However, regarding meta 2.2 (Figure 5d), the retreat in L3 movement was capable of generating a much more open catalytic site cavity, making CYS472 more available in this metastable state when compared to the other inhibited conformations.

In addition, to observe clearly how inhibition affects CYS472 availability in terms of L1 and L3 movement, Figure 6 shows the most populated meta of each system side by side with chain B monomer of PDB 6F7I,^29^ an inhibited crystal of MALT1 which contains a fully crystallized Loop 3. From Figures 6B and 6C it is possible to observe that both crystal and the most populated inhibited metastate present an L1 and L3 bent toward the catalytic site, which makes the catalytic cysteine more unavailable, as can be seen in the surface representation of both structures. In this sense, the observed comparison shows an important agreement between the theoretical collected structure and the experimentally obtained MALT1 inhibited crystal. Furthermore, a previous *in silico* study developed by Zhang et al.^28^ also emphasized that L3 has an important role in covering the catalytic cysteine in the MALT1 inactive state, showing good agreement with the presented theoretical findings in this study.

**Figure 6:**
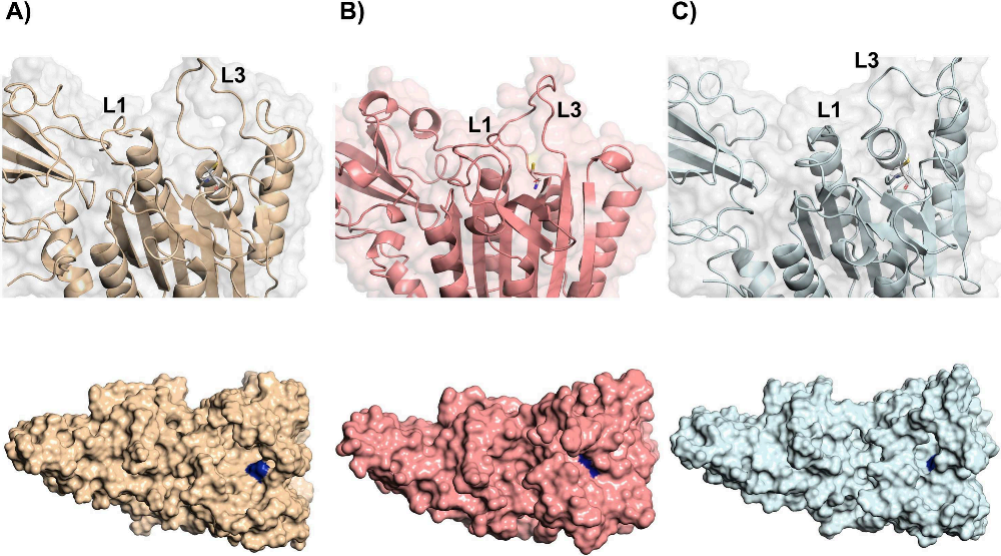
Cartoon and surface representations focusing on the catalytic site of MALT1 of (A) meta 1.1, (B) chain B of PDB 6F7I, and (C) meta 2.1. In all structures, L1 and L3 are annotated, where catalytic cysteine is shown as sticks, and in surface representation, the catalytic cysteine is colored in dark blue.

Despite the important agreement observed between the theoretical structures and the experimental structure, it is important to mention that by examining all the available MALT1 crystal structures in RCSB PDB^64^ (majorly human MALT1), it seems that Loop 1 shows virtually the same conformation across structures, presenting no relevant mobility. In addition, the investigation performed by Wallerstein et al.^30^ showed from experimental backbone relaxation data of human MALT1 that Loop 3 mobility is much more relevant than Loop 1 mobility. Furthermore, from Figure 1 it is possible to observe that Loop 1 (residues 360 to 367 in mouse MALT1) presents two mismatch residues with human MALT1 (S360N and W362R); thus, this difference might imply different dynamics of Loop 1 between human and mouse MALT1 as well. Therefore, the mobility of Loop 1 observed until now for mouse MALT1, when compared with previous human MALT1 findings, shows that the described Loop 1 movements generalization to all MALT1 proteins should be taken with care.

Now, for a better comprehension of the effects of L1 and L3 movements in the catalytic site cavity size and also to allow a better comparison between systems, the catalytic site cavity volumes of each selected structure were calculated, where the values are shown in Table 1. For the calculation, the CavityPlus^65,66^ web server was used.

**Table 1:** Calculated Catalytic Site Cavity Volumes for Each Monomer Metastable State of Each System

	volume (Å^3^)	
metastate	System 1	System 2
meta 1	2863.50	601.00
meta 2	1858.00	1729.50
meta 3	1454.25	536.50

In Table 1, it is observed that all selected conformations of system 1 presented a high catalytic site cavity volume. For meta 1.1, a volume of 2863.50 Å^3^ was observed, for meta 1.2, 1858.00 Å^3^, and for meta 1.3, 1454.25 Å^3^. One important thing to notice is that meta 1.1 (Figure 4a) presented a substantially higher volume than meta 1.2 and 1.3, which is in accordance with the fact that for this metastable state, it was observed a much more pronounced L1 bending toward the opposite direction of the catalytic site. In addition, the L3 opening in the referred metastate is also more pronounced than in the other metastable states.

Regarding system 2, it was possible to observe from Table 1 that when there is an L1 and L3 movement toward the catalytic site (case of meta 2.1 and 2.3 of system 2, Figures 5a and 5e), the volume of the cavity substantially decreases when compared to metastable states that do not present those movements (like system 1 metastates). Volumes of 601.00 and 536.50 Å^3^ were observed for meta 2.1 and 2.3 of system 2, respectively. On the other hand, when a retreat is observed in L3, which is the case of meta 2.2 of system 2 (Figure 5c), it was observed that the cavity volume is similar to the ones where CYS472 is more available for substrate binding, which is the noninhibited system. For the meta 2.2 structure of system 2, a volume of 1729.50 Å^3^ was observed. Therefore, since meta 2.2 of system 2 was not capable of generating an appropriate conformation for the L1 and L3 left portions to be closer in space, a high cavity volume was observed.

Remembering the performing population analysis, it is possible to achieve an interesting conclusion. For system 1, 100% of the labeled conformations present a high cavity volume, appropriate for CYS472 availability for substrate binding. Now, after the positioning of an allosteric inhibitor, it was possible to notice that 79.9% of the labeled conformations presented a much lower cavity volume, and only 20.1% of the conformations presented high cavity volume, similar to the noninhibited system.

Therefore, the allosteric inhibitor was able to stabilize a great majority of much less available CYS472 conformations, which is crucial for MALT1 inactivity.^15,25^ Hence, the results presented until now show that the allosteric inhibition of MALT1 performs relevant L1 and L3 movements in order to decrease the catalytic site volume, where statistically, it occurs on the great majority of the conformations. In this scenario, with the current information, it is now possible to evaluate CYS472 availability in more appropriate terms through docking analysis of the dimeric form, where MALT1 attains its catalytically active state.

### The Role of Monomeric Metastable States in MALT1 Dimer Formation

One crucial step for MALT1 activation resides in its dimerization, from which an active complex can be formed and its biochemical activity can be performed.^5,25,30^ Together with the complex formation, the dimerization process helps stabilize an active paracaspase conformation,^5,15,30^ in which CYS472 is more available for substrate binding. In this sense, it is much more appropriate to evaluate the availability of CYS472 in the formed dimer, where the catalytic site conformation is presented after the activation procedure in which MALT1 is submitted.

In this scenario, protein–protein docking was performed for all collected metastable structures in order to build dimers. In total, six dimers were built for each system; three dimers were formed by combining the same metastable monomeric unit, and three dimers were formed by combining the metastable monomeric units of its respective system, as shown in Figure 7. Therefore, energy score data and catalytic cavity volumes are shown in Table 2, where, since they are related to the built dimer, the catalytic site cavity volume is shown as a sum of the monomeric unity cavity volumes. In addition, Table 2 also shows the cavity volume of the crystal dimer PDB 6F7I.

**Figure 7:**
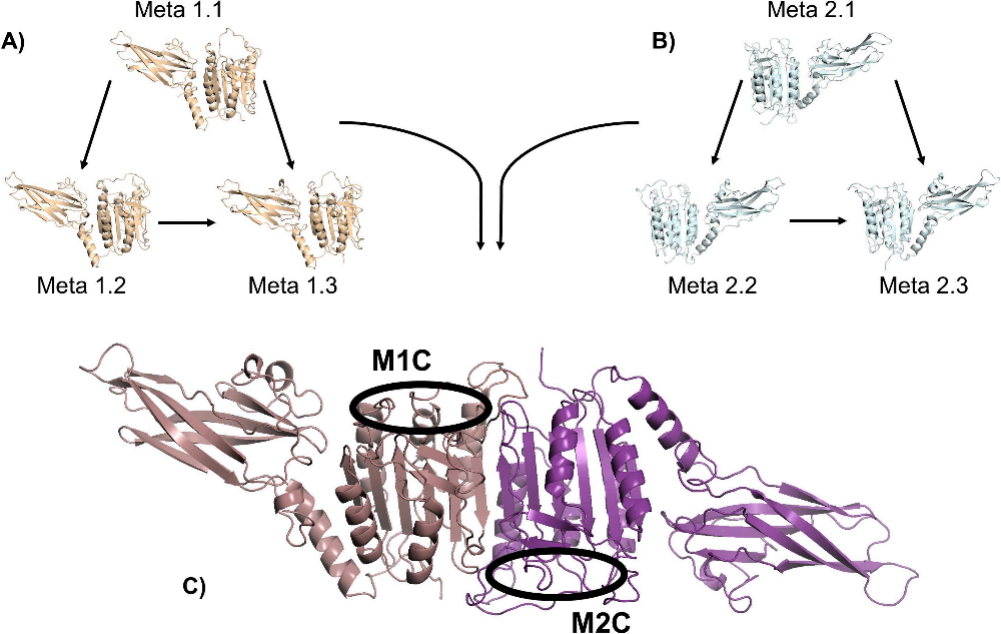
Scheme showing how dimers were constructed and the cavity volumes accounted for in the further tables. The dimers were built from the combination of metastable monomeric units, combining one meta monomer with itself in its respective systems, and also combining (A) one meta monomer of system 1 with other meta monomers of the same system to build system 1 dimers and (B) one meta monomer of system 2 with other meta monomers of the same system to build system 2 dimers. (C) Constructed dimers, where two catalytic sites are presented, M1C being the monomeric unity 1 cavity and M2C the monomeric unity 2 cavity.

**Table 2:** Calculated Protein–Protein Docking Energy Scores for Dimer Formation and the Catalytic Site Cavity Volume of Each Obtained Dimer^a^

meta combination	energy scores	cavity volume (Å^3^)
1.1 and 1.1	–3834.0	5781.34
1.2 and 1.2	–3937.9	3793.89
1.3 and 1.3	–4015.7	3109.70
1.1 and 1.2	–3987.4	4778.69
1.1 and 1.3	–3824.8	4439.28
1.2 and 1.3	–3851.4	3457.43
2.1 and 2.1	–3892.2	1461.88
2.2 and 2.2	–3919.8	3619.09
2.3 and 2.3	–4381.3	860.57
2.1 and 2.2	–3956.4	2541.49
2.1 and 2.3	–4503.4	1153.80
2.2 and 2.3	–3986.4	2241.05
crystal dimer (PDB: 6F7I)^29^	–	1826.13

a The shown cavity volumes are the result of the sum of the cavity volumes of the monomeric units (cavity volume = M1C + M2C), where the monomeric cavities can be seen in Figure 7, adding in the table the cavity volume of crystal dimer PDB 6F7I. The dimers were built in pairs, where the monomer of one metastable state was docked with itself and with the other metastable monomers of the same system.

Average energy scores of −3908.6 kcal/mol for system 1 and −4106.6 kcal/mol for system 2 were observed, showing that both the noninhibited and the inhibited systems were capable of forming stable dimers with similar energy scores. Regarding the catalytic site cavity volumes of the constructed dimers, it was observed a small change when compared to the monomers that formed the dimers (divide Table 2 volume data by 2 and compare with Table 1 data); however, the volumes were still almost the same, showing that the dimer formation did not have a great influence on the catalytic site cavity volume. Moreover, it is important to mention that the crystal dimer (PDB: 6F7I) presents a cavity volume value closer to the inhibited dimers, showing good agreement between our constructed structures and the experimentally obtained inhibited crystal.

From the constructed dimers, an appropriate analysis of CYS472 availability could be possible once the dimerization step is needed for MALT1 activation through catalytic site cavity stabilization. In this sense, using the built dimers, it was possible to move further and evaluate CYS472 availability with a substrate mimic.

### Analyzing CYS472 Availability in the Constructed Dimers through Substrate Mimic Covalent Docking

For the CYS472 availability analysis, the Z-VRPR-fmk irreversible covalent inhibitor was used to mimic the substrate in the binding site evaluation. Irreversible covalent inhibitors, such as the peptidic inhibitor Z-VRPR-fmk, compete with the substrate once it covalently binds to the sulfhydryl group of cysteine in MALT1,^5,25^ making it an appropriate substrate mimic to evaluate the effects of the catalytic site cavity volume in CYS472 availability. Therefore, Z-VRPR-fmk was used for covalent docking in the catalytic site of the built metastable structure dimers, where the main goal here is to evaluate the binding affinity in terms of docking energy and correlate it with the cavity volume.

The docking energies are shown in Table 3 as the docking energies in the dimeric form. The obtained energy values are in accordance with the hypothesized effect, where much higher energies were obtained for those dimers formed with at least one monomeric unity with a small cavity volume, as discussed in the previous section. While the energy of system 1, which all dimer structures presented larger catalytic site cavity volumes, is kept between −2.5 and 1.1 kcal/mol, the energy of the dimers that contain at least one monomeric conformation belonging to meta 2.1 and meta 2.3 in system 2, which presented much smaller cavity volumes, is kept between 19.6 and 84.4 kcal/mol, respectively. In addition, the only dimer in system 2 that presented a small energy, equivalent to the ones obtained for the active dimers in system 1, was the dimer formed by two meta 2.2 monomeric units, which presented a larger cavity volume (on the same scale as the ones observed in system 1), presenting an energy of −3.3 kcal/mol.

**Table 3:** Calculated Covalent Docking Energies between Z-VRPR-fmk Peptidic Inhibitor (Substrate Mimic) and Built Dimers from Collected Metastable Structures of MALT1

meta combination	energy (kcal/mol)
1.1 and 1.1	–2.5
1.2 and 1.2	1.1
1.3 and 1.3	–1.3
1.1 and 1.2	–0.6
1.1 and 1.3	–2.1
1.2 and 1.3	–0.2
2.1 and 2.1	43.2
2.2 and 2.2	–3.3
2.3 and 2.3	84.4
2.1 and 2.2	19.6
2.1 and 2.3	62.7
2.2 and 2.3	40.1

From the constructed metastable monomeric combinations to form dimers and from the known population distribution of the metastable monomers in both systems, it was also possible to obtain valuable information about the population of the active and inactive inhibited dimers. The proportion of metastable monomers meta 2.1, 2.2, and 2.3 follows as 58.7%/20.2%/21.1% of the labeled conformations, respectively. In that regard, considering that the formation of dimers is driven only by its population size, a quick calculation can be performed, where the population of a formed dimer follows *P* = *M*1_*p*_ × *M*2_*p*_, where *P* is the dimer population, *M*1_*p*_ is the population proportion of the first monomeric unit, and *M*2_*p*_ is the population of the second monomeric unit. Hence, Table 4 shows the calculated populations of the constructed dimers, where the population of the dimers formed by monomeric units M1 and M2 are multiplied by two, once the dimer formed by M1 with M2 and M2 with M1 is the same dimer.

**Table 4:** Calculated Population for Each Constructed Dimer of System 2, Where the Population Calculation Follows *P* = *M*1_*p*_ × *M*2_*p*_ ^a^

meta combination	population proportion
2.1 and 2.1	34.5%
2.2 and 2.2	4.1%
2.3 and 2.3	4.5%
2.1 and 2.2	23.8%
2.1 and 2.3	24.8%
2.2 and 2.3	8.6%
total	∼100%

a In addition, since the constructed dimer of monomeric units M1 and M2 is the same as the one constructed from M2 and M1, their population was multiplied by two in order to correctly assign its proportion on the investigated ensemble.

It was possible to observe from the obtained results in Table 4 that the dimers formed by at least one monomeric unit of meta 2.1 represent 83.1% of the total population. When adding to this number the proportion of dimers that are formed by at least one monomeric unit of meta 2.3 and no monomeric unit of meta 2.1, this proportion increases to 96.2%. Therefore, since only the dimer formed by meta 2.2 and meta 2.2 presented a docking energy consistent with an active dimer, the obtained results show that MLT-748 allosteric inhibition is capable of inactivating 96.2% of the MALT1 dimers, showing its great potency, which is in accordance with previously reported experimental data in literature.^29^ In this sense, the computational results presented in this work strongly support the experimental data available for MLT-748.

In this scenario, the obtained results are of major importance, showing that the allosteric inhibition of MALT1 through the MLT-748 inhibitor occurs by promoting important structural changes in L1 and L3 conformations, movements that can be seen in Figure 8, which shows a scheme of MALT1 inhibition through the MLT-748 allosteric inhibitor. It was noticed that a movement of both loops toward the catalytic site cavity direction had a crucial impact on the cavity volume and CYS472 availability for substrate binding. Therefore, the obtained results show valuable information on MALT1 allosteric inhibition and act as objective criteria for observation when designing new potential MALT1 allosteric inhibitors.

**Figure 8:**
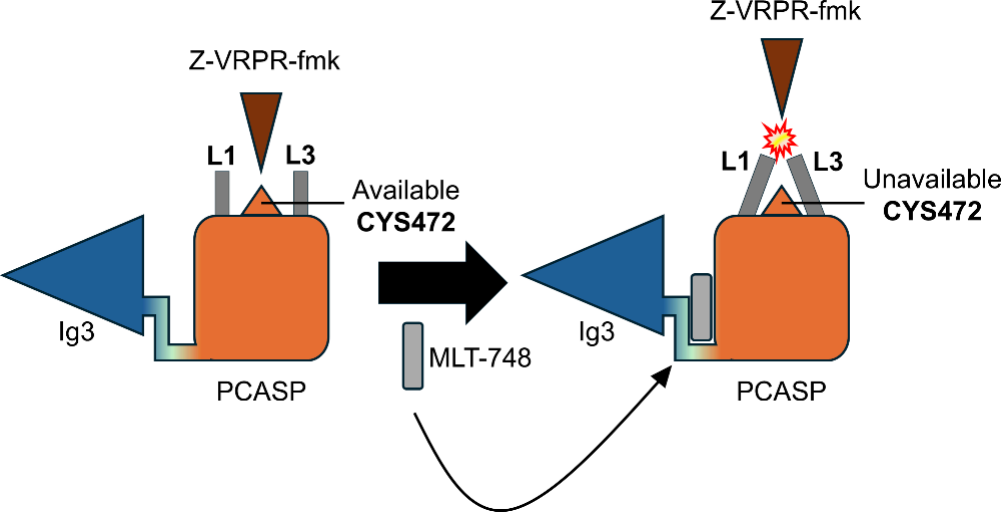
Allosteric inhibition scheme of MALT1 protein. In the figure, PCASP refers to the paracaspase domain of MALT1, and Ig3 refers to the immunoglobulin domain of MALT1.

## Conclusions

The present work shows that from an entirely theoretical method, it was possible to achieve important information about MALT1 allosteric inhibition and to investigate other intrinsically disordered proteins.^17^ This was possible by using biased MD simulations, which allow access to metastable states, and by using machine learning techniques, such as DeepTICA analysis, which allow the identification and characterization of the accessed metastable states. In addition, the present work sums up other works in the literature to show the versatile usage of neural networks in the chemistry field, more specifically, in computational drug design. When traditional techniques, such as MD simulations and molecular docking, are combined with modern machine learning, such as neural networks, the result is a powerful protocol to unravel complex protein motions and inhibition patterns.

From the presented results, it was shown that after positioning the allosteric inhibitor in the MALT1 protein different monomeric metastable states were stabilized, modifying the dynamical behavior of this complex protein. Moreover, it was observed that the allosteric inhibition of MALT1 through the MLT-748 inhibitor usage occurs from profound conformational rearrangements in the catalytic site. After allosteric inhibition, a trend of Loop 1 and Loop 3 movement toward the catalytic site was verified, reducing its cavity volume and CYS472 availability. The mentioned results are strongly supported by the inspection of representative structures and by docking results with a substrate mimic in the constructed dimers, showing that in the inhibited scenario the covalent binding of the substrate mimic is substantially hampered. The obtained results were also capable of showing the statistical nature of such conformational rearrangements responsible for inactivating MALT1. It was observed that after allosteric inhibition 96.2% of the obtained dimers achieved an inactive conformation. This result strongly supports the previously reported experimental potency of the MLT-748 allosteric inhibition. However, from the comparison between the observed loop motions and the available crystal structures, it is important to warn readers to take care when generalizing the obtained results for all MALT1 proteins, emphasizing that these findings are related to the mouse MALT1 construct, which presents 93% homology with the human MALT1.

In that regard, such information is of great value and can be used as important metrics for the computational design of new MALT1 allosteric inhibitors. While the analysis of the catalytic site cavity volume can serve as an objective criterion for determining the viability of a promising new allosteric inhibitor, the population statistical data can be used as a metric to verify the potency of a new candidate. Finally, we hope to provide new insights into MALT1 allosteric inhibition from this study, as we believe that the theoretical protocol developed here can be extended to other intrinsically disordered proteins (IDPs), which pose significant challenges for structural validation. This is particularly relevant because the three-dimensional native structure of a protein lies at the core of the structure–function paradigm. The unusual behavior of such proteins has long contributed to the underestimation of protein disorder, highlighting the difficulty in fully understanding their mechanisms of action and, consequently, in designing new inhibitors.

## Supplementary Material



## Data Availability

GROMACS is available at https://www.gromacs.org/. PLUMED is available at https://www.plumed.org/. Hbonds descriptors, DeepTICA values and conformation labels, neural network trained weights, representative metastable conformations, COLVAR files, simulation inputs, and data analysis codes are available at https://zenodo.org/records/17088787?token=eyJhbGciOiJIUzUxMiJ9.eyJpZCI6IjJkOTA0ZWNhLWZjYWYtNDM4NS1hMjFmLWM4MjI0NDkwOTg5MiIsImRhdGEiOnt9LCJyYW5kb20iOiI1ZmUyMmViZjVjOThkMjVlMzRiZDU1YWViYzhiNDBiMCJ9.KG2iJIl_-wh-y1YN1nUwPP7d2uXNqteAs8Z3estwYM4WsxiONCLxe0cxZGJl4n-W17DmRFY8IN8xER36Wt7tJw.

## Notes

Additional DeepTICA figures, bias convergence profiles, RMSD analysis of the unbiased and biased systems, additional information about the constructed neural network models, and external validation of the used ANN classifier in the DeepTICA space (PDF)
