# Supplementary material for: Decoding Allosteric Inhibition in MALT1: The Hidden Role of Conformational Plasticity in Metastable States via Biased MD and Deep Learning
Source: J Phys Chem B. 2026 Jan 20;130(4):1182–96. doi: 10.1021/acs.jpcb.5c07665 (PMC12862816; doi:10.1021/acs.jpcb.5c07665)
Supplement: Supplementary file 1 [file jp5c07665_si_002.pdf]

SUPPORTING INFORMATION: Decoding Allosteric Inhibition in MALT1: The Hidden Role of Conformational Plasticity in Metastable States via Biased MD and Deep Learning

Rodrigo M. Santos,† Tainúh M. R. Santos,† and Teodorico C. Ramalho*,†,‡

†*Laboratory of Molecular Modelling, Department of Chemistry, Federal University of Lavras, Lavras 37200-000, Minas Gerais, Brazil*

‡*Centre for Basic and Applied Research, Faculty of Informatics and Management, University of Hradec Králové, Hradec Králové 500 03, Czech Republic*

E-mail: rodrigomancini4@gmail.com,tainah-martins@hotmail.com,teo@ufla.br

SI. 1 - Biased MD simulations bias convergence

It is possible to observe from Figure S1 a converged bias after 50 ns of the simulation, making the slicing after this time an appropriate choice in order to use the computed descriptors for further DeepTICA analysis.

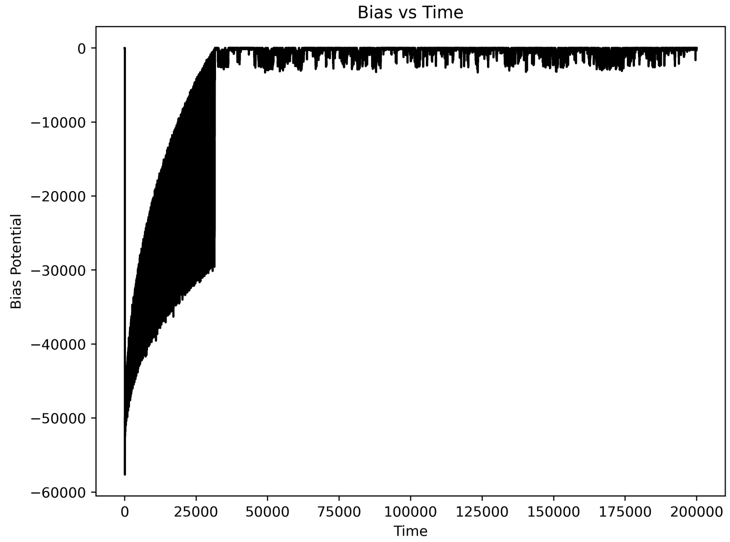

(a)

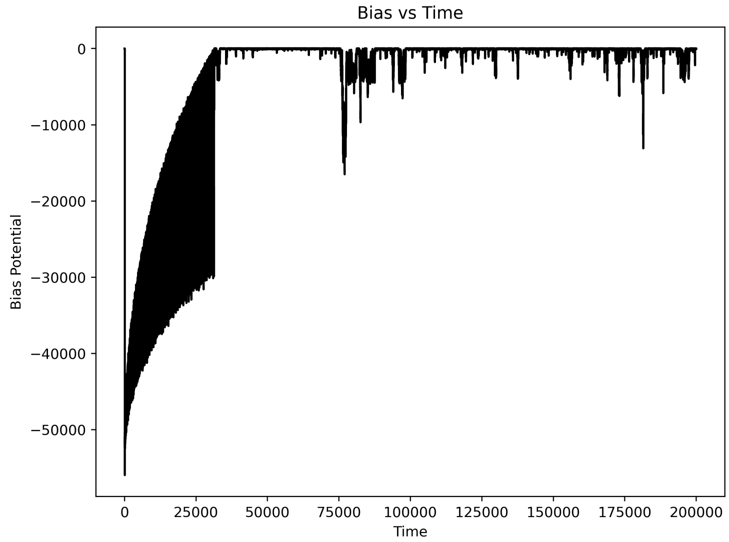

(b)

Figure S1: Bias profile over biased simulation time for a) system 1 and b) system 2.

SI. 2 - Unbiased MD RMSD analysis

From the obtained replicas, unbiased MD simulation trajectories were analyzed in terms of their backbone atoms RMSD, taking as reference the simulation initial structure of each replica, with RMSD profile shown in Figure S2. From this evaluation, it was possible to observe the evolution of both the non-inhibited system (system 1) and inhibited system (system 2). This information can be of great value in order to understand further analysis such as the comprehension of metastable states distribution and characterization.

From Figure S2a, it was observed that in system 1 MALT1 protein presented an RMSD average of 3.6 Å for replica 1, 2.3 Å for replica 2, and 1.9 Å for replica 3, with standard deviations of 0.9 Å, 0.3 Å, and 0.2 Å, respectively. In addition, for the replicas of system 1, it was obtained an overall RMSD average of 2.6 Å and an overall standard deviation of 0.7 Å were obtained. Now, from Figure S2b, it was observed that in system 2, MALT1 protein presented an RMSD average of 2.5 Å for replica 1, 2.8 Å for replica 2, and 2.2 Å for replica 3, with standard deviations of 0.3 Å, 0.4 Å, and 0.3 Å, respectively. In this sense, for the replicas of system 2, it was obtained an overall RMSD average of 2.5 Å and an overall standard deviation of 0.3 Å were obtained.

Therefore, it was possible to observe that after positioning the MLT-748 allosteric inhibitor in MALT1 protein, more stable MALT1 conformations were achieved, indicated by a considerable reduction of overall standard deviation between the simulated replicas when comparing systems 1 and 2. In addition, the higher conformational plasticity in the non-inhibited system is demonstrated by the capability of non-inhibited MALT1 in producing slightly different MD evolutions, like the one observed for the first replica when compared to the other replicas. In this sense, the observed evolutions for system 2 show MALT1 with fewer degrees of freedom when compared to non-inhibited MALT1, which is expected once the positioning of the allosteric inhibitor hampers conformational rearrangements.

Now, regarding specifically system 1, its first replica showed a considerable difference when compared to the others. Therefore, an RMSF analysis was performed for all the

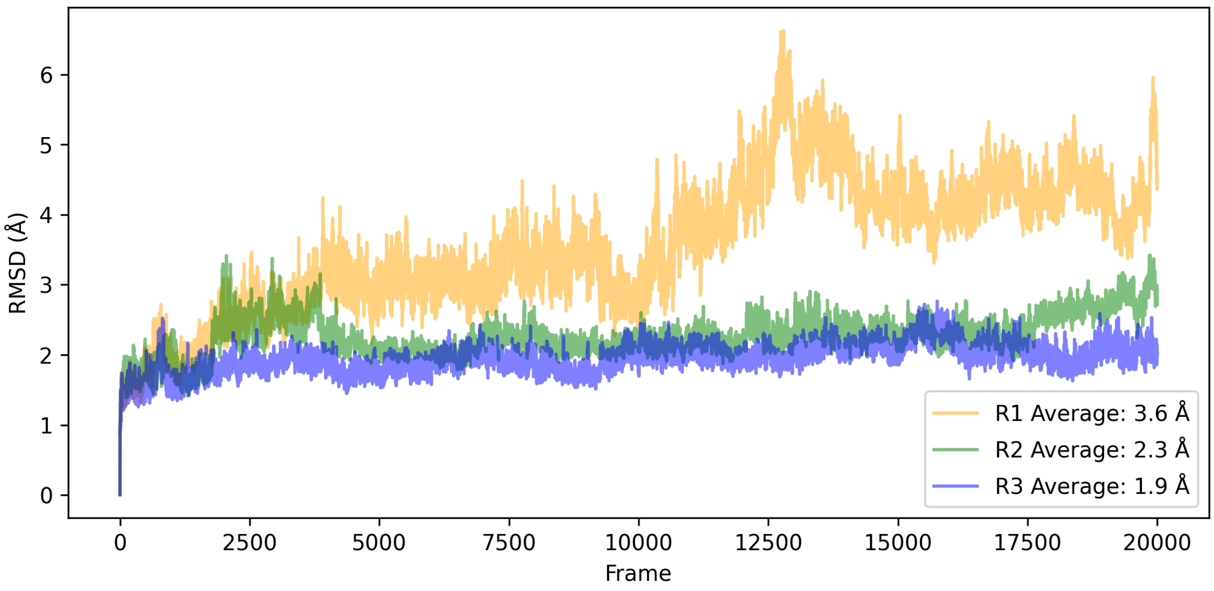

(a)

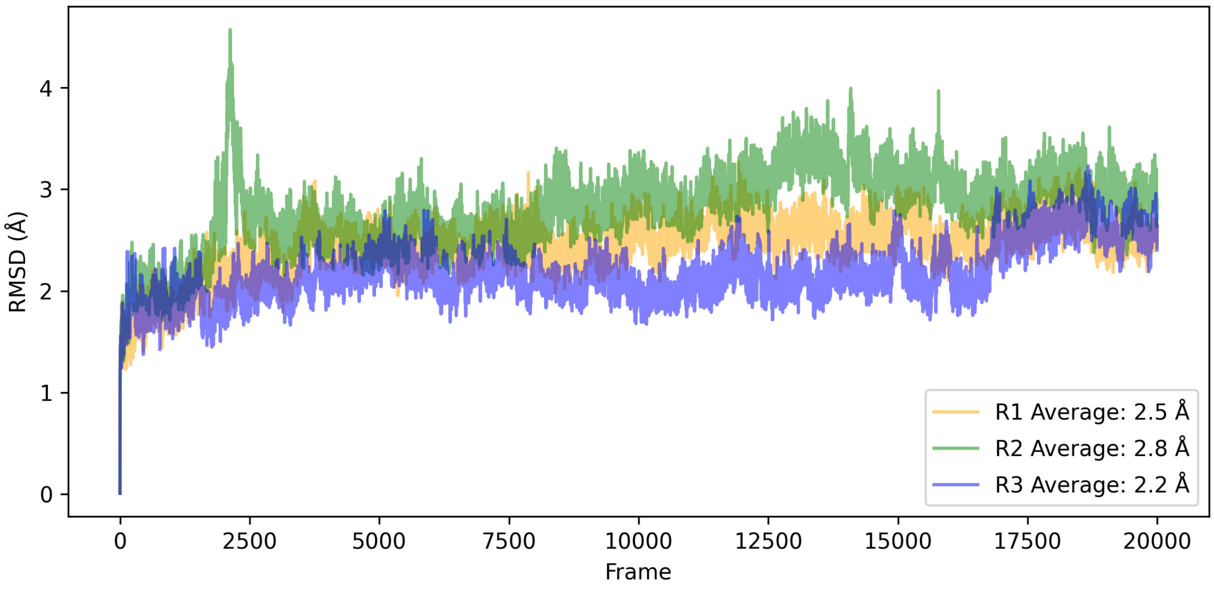

(b)

Figure S2: RMSD profile of the unbiased MD simulations trajectories for a) replicas of system 1 and b) replicas of system 2.

replicas of system 1 in order to observe which residues fluctuate the most, being capable of generating a different MD evolution. In this sense, Figure S3 shows the computed RMSF for each residue of each replica.

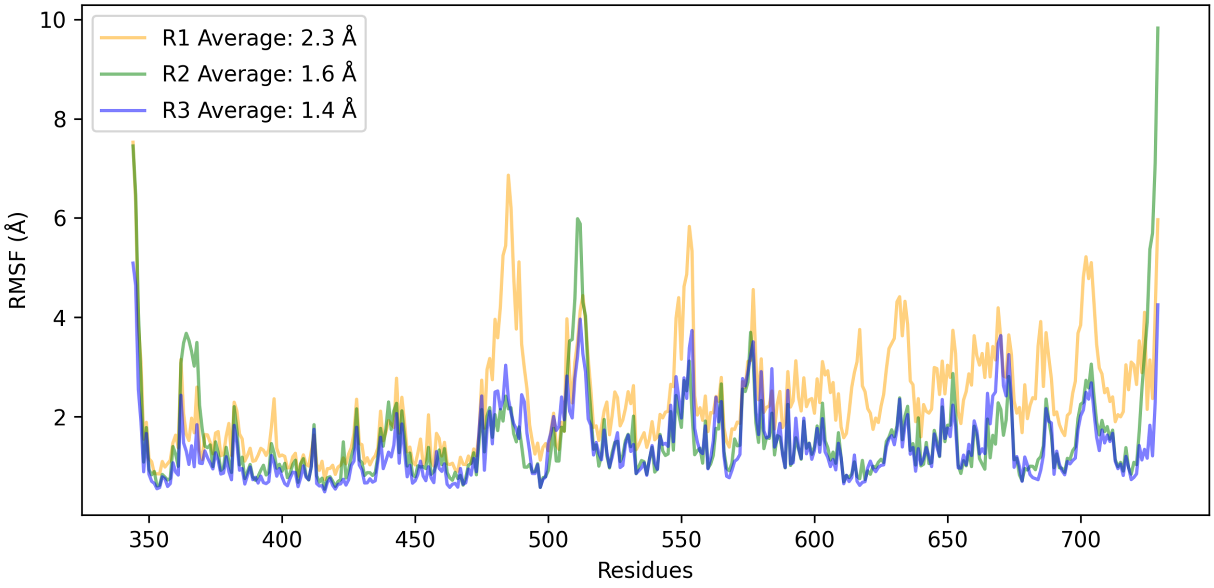

Figure S3: Calculated RMSF for each residue of each of the unbiased MD replicas of system 1.

From Figure S3 it is possible to observe a very similar profile and RMSF values for both replicas 2 and 3, an expected behavior once the RMSD profiles of both replicas showed a very similar evolution. However, when observing replica 1's RMSF profile, it was possible to observe some differences in some specific residue intervals. By analyzing the RMSF profile of replica 1, four intervals were observed as varying the most with respect to replicas 2 and 3. The intervals are residues 475-490 containing Loop 2 chain located at the MALT1 dimer interface (1); residues 540-560 containing β sheet 6 and α helix 5, both located at the MALT1 dimer interface (2); residues 600-650 (3); and residues 690-720 (4), both chains located at the Ig3 domain of MALT1.

In this sense, it was observed that none of the observed RMSF differences between replica 1 and replicas 2 and 3 were related to the catalytically important loops. Hence, the verified chains increased fluctuations in replica 1 explain the observed evolution verified through the RMSD profile and reinforce the need for the usage of biased MD simulations for an improving conformational exploration of MALT1.

SI. 3 - DeepTICA Analysis Additional Information

The obtained conformational landscapes for systems 1 and 2 with the associated computed free energy surface (FES) are shown in Figure S4.

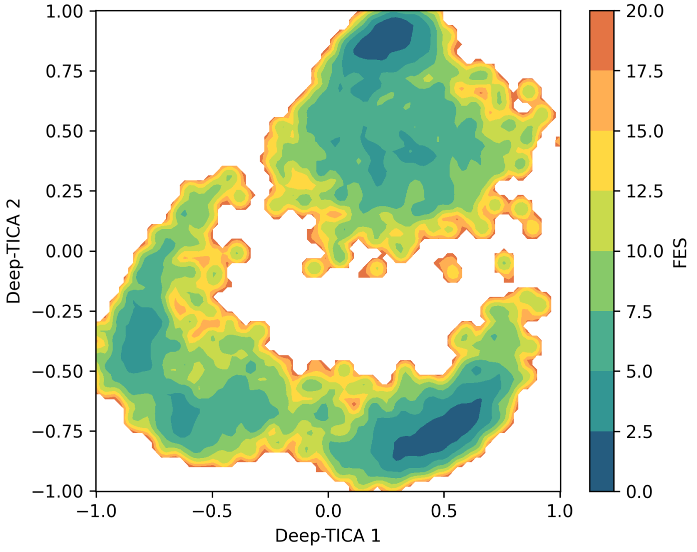

(a)

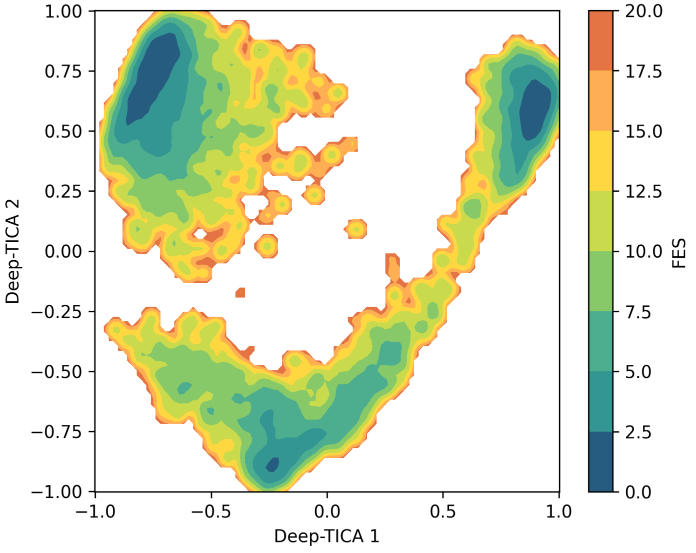

(b)

Figure S4: DeepTICA conformational landscape plot with the associated computed free energy surface (FES), displayed in a colorbar, of a) system 1 and b) system 2.

From Figure S5, it is possible to observe the learning curves during training of the artificial neural network used for generating the DeepTICA conformational landscapes.

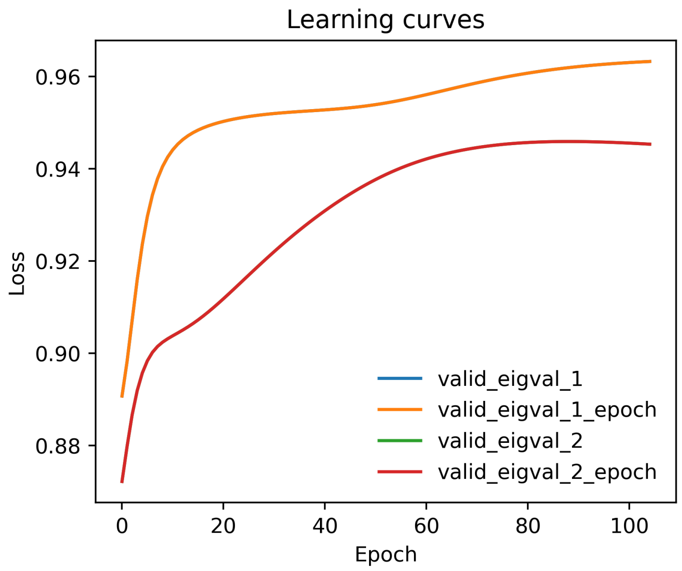

(a)

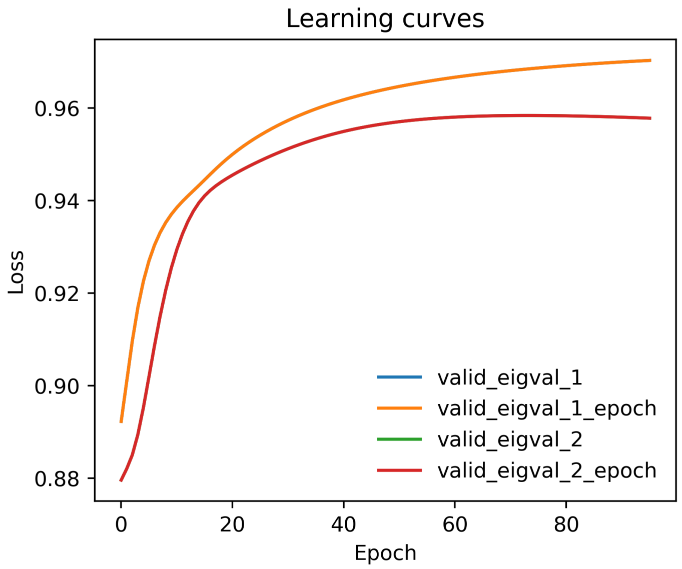

(b)

Figure S5: DeepTICA learning curves obtained during training of a) system 1 and b) system 2.

SI.4 - MLP Classifier Additional Information

The MLP Classifier took as inputs the same hydrogen bond distances that were used to build the DeepTICA conformational landscape; therefore, the architecture of the classifier, shown in Figure S6, used one input layer, three hidden layers, and one output layer, with the following number of neurons [602,100,100,100,3]. From this architecture, 80803 weights and biases were trained (see Figure S7 for loss and accuracy profiles during training), where 1000 samples were separated from the dataset to be tested after training, and 20% of the trainable dataset was separated for validation during training. The hidden layers used ReLU as an activation function, and Softmax was used as output layer activation function. For the training, the ADAM optimizer was used with the categorical crossentropy loss function, where prediction accuracy was evaluated during training.

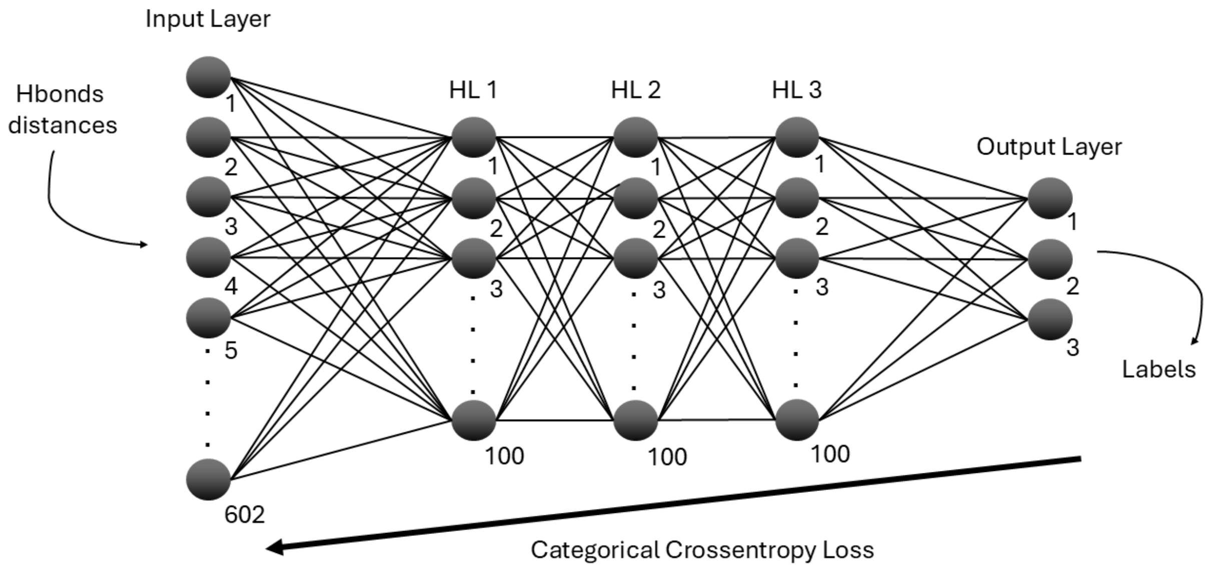

Figure S6: MLP classifier architecture, where HL are the hidden layers, and the grey circles are the neurons.

The loss and accuracy over epochs can be seen in Figure S7, where it was possible to observe that both systems achieved a very small loss and a 100% accuracy during training.

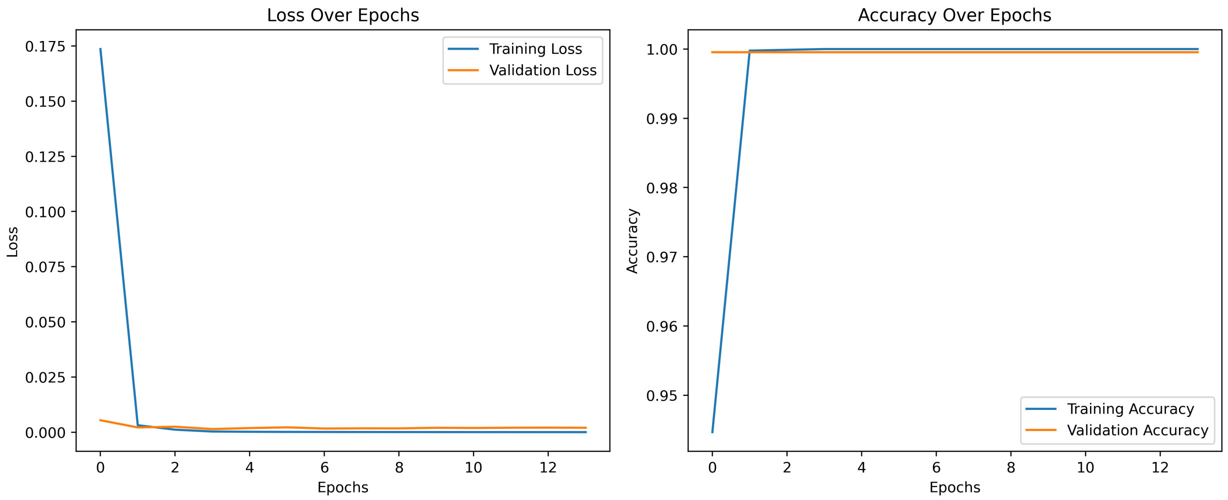

(a)

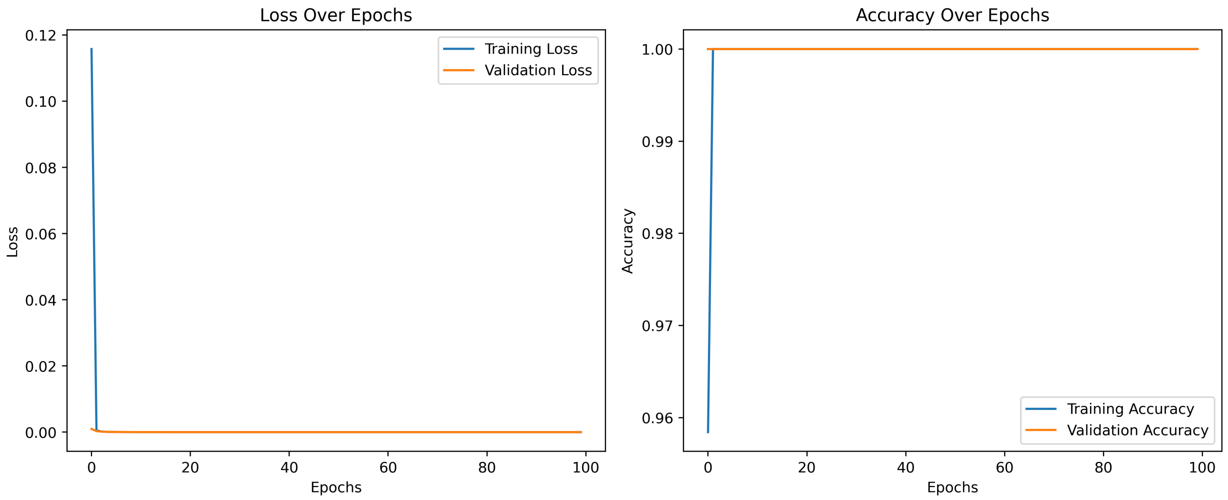

(b)

Figure S7: Loss and accuracy during training of the built ANNs for labeling validation and metastable equivalence between systems verification for a) system 1,and b) system 2.

Now, Figure S8 shows the ANN built from system 1 data prediction of the separated system 1 data and also the prediction of the separated system 2 data. It is possible to observe a perfect prediction of the separated system 1 data; however, a poor prediction of system 2 separated data reinforces that the observed metastable states are not equivalent between systems.

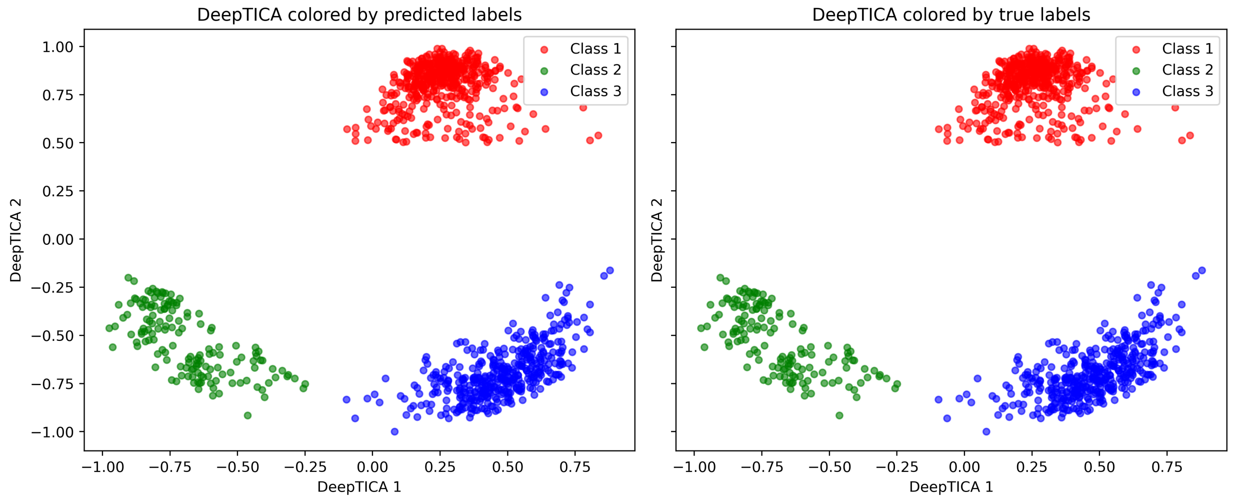

(a)

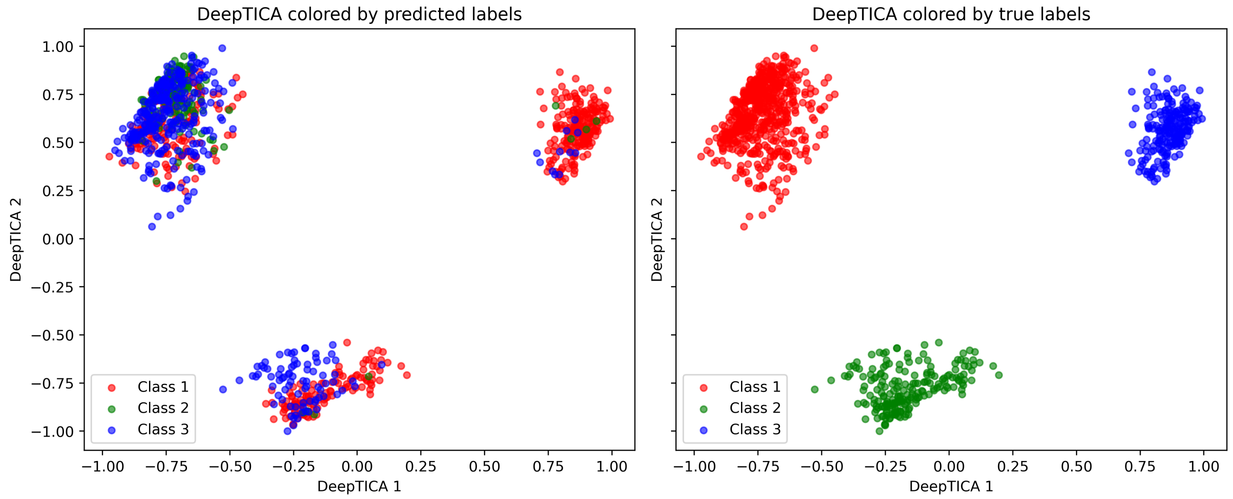

(b)

Figure S8: a) Unseen system 1 data predicted by system 1 ANN, and b) unseen system 2 data predicted by system 1 ANN. In addition, the samples are shown in the DeepTICA 2D space.

Moving further, Figure S9 shows the ANN built from system 2 data prediction of the separated system 2 data and also the prediction of the separated system 1 data. It is possible to observe a perfect prediction of the separated system 2 data; however, a poor prediction of system 1 separated data. This provides additional evidence that the observed metastable states are not equivalent between systems.

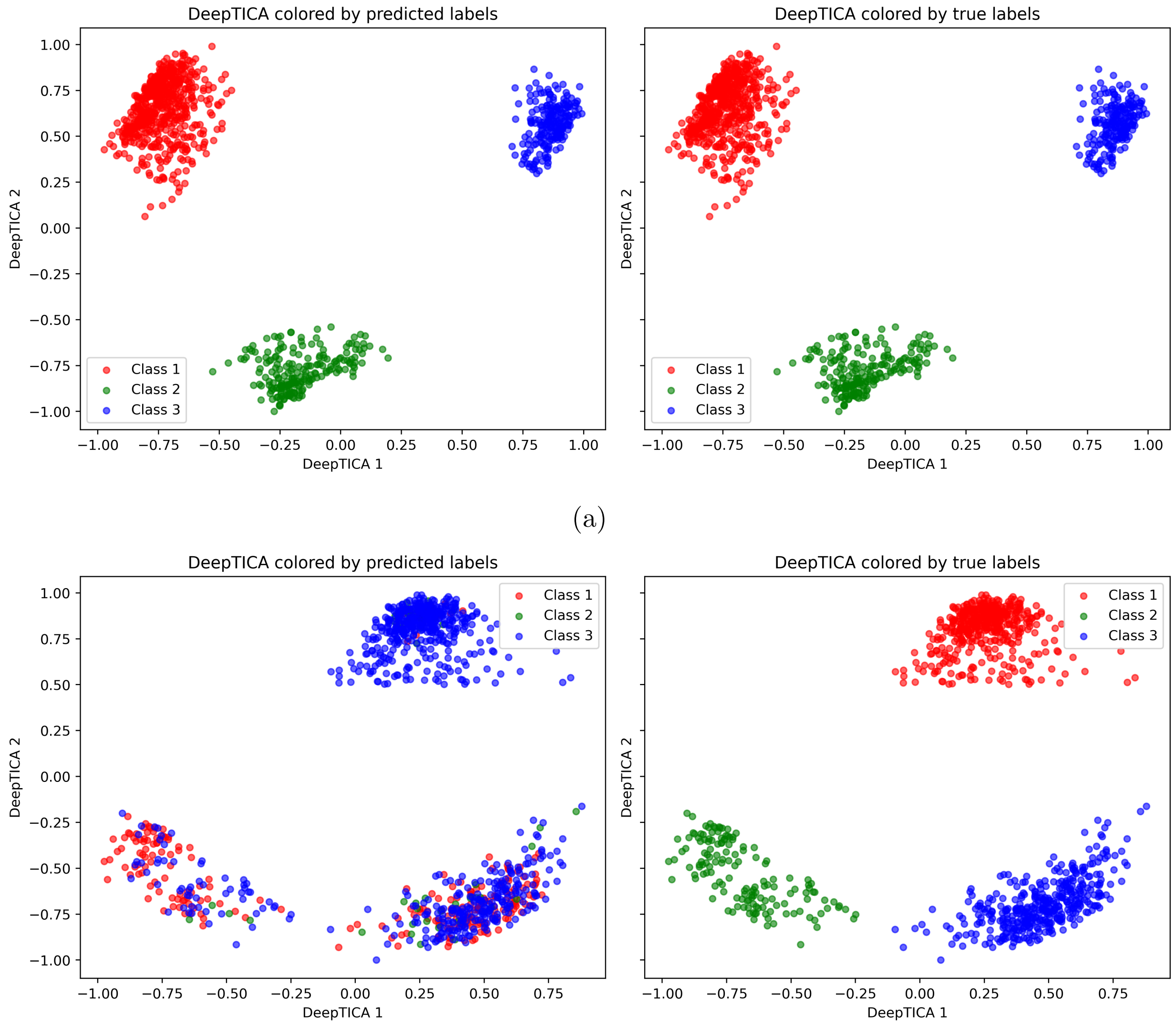

(a)

(b)

Figure S9: a) Unseen system 2 data predicted by system 2 ANN, and b) unseen system 1 data predicted by system 2 ANN. In addition, the samples are shown in the DeepTICA 2D space.

SI.5 - Biased MD RMSD analysis

In order to observe the biased MD evolution, the backbone RMSD of the obtained structures was computed, taking as reference the crystal structure of *mus musculus* MALT1 (PDB: 3V4L).[1] This calculation can be of great value once the mentioned crystal is in an active state.[1,2]

In this scenario, Figure S10 shows the calculated backbone RMSDs for both systems. For system 1, an average RMSD of 2.9 Åwas obtained, and for system 2, an average RMSD of 4.1 Å. Therefore, the computed RMSD for system 2 is considerably higher than the one computed for system 1, showing that the accessed structures of system 2 deviate more from the crystal active structure. Therefore, the presence of an allosteric inhibitor is capable of performing considerable structural modifications, being part of MALT1 inactivation.

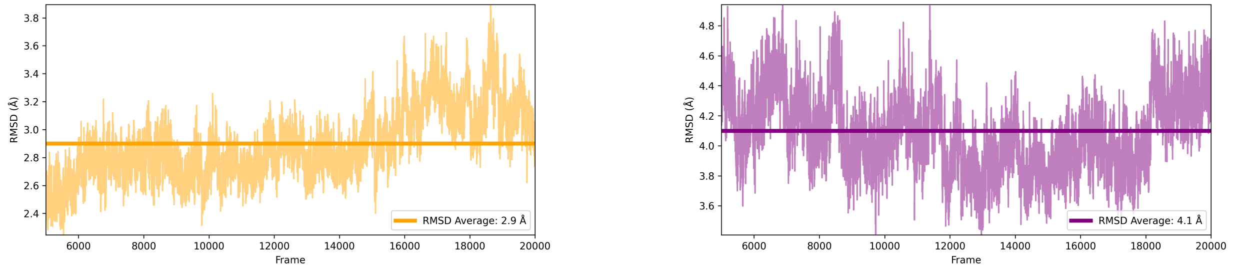

(a) (b)

Figure S10: RMSD profile of the accessed MALT1 conformations with respect to *mus musculus* MALT1 active structure (PDB: 3V4L).[1]

References

(1) Wiesmann, C.; Leder, L.; Blank, J.; Bernardi, A.; Melkko, S.; Decock, A.; D'Arcy, A.; Villard, F.; Erbel, P.; Hughes, N.; Freuler, F.; Nikolay, R.; Alves, J.; Bornancin, F.; Renatus, M. Structural Determinants of MALT1 Protease Activity. *Journal of Molecular Biology* **2012**, *419*, 4–21.

(2) Quancard, J. et al. An allosteric MALT1 inhibitor is a molecular corrector rescuing function in an immunodeficient patient. *Nat Chem Biol* **2019**, *15*, 304–313.
